# Auditory thresholds compatible with optimal speech reception likely evolved before the human-chimpanzee split

**DOI:** 10.1038/s41598-023-47778-2

**Published:** 2023-11-25

**Authors:** Alexander Stoessel, Romain David, Matthias Bornitz, Steffen Ossmann, Marcus Neudert

**Affiliations:** 1 Institute of Zoology and Evolutionary Research, Friedrich Schiller University Jena, Erbertstr. 1, 07743 Jena, Germany; 2 Max Planck Institute for Evolutionary Anthropology, 04103 Leipzig, Germany; 3 Centre for Human Evolution Research, The Natural History Museum, Cromwell Rd, South Kensington, London, SW7 5BD UK; 4 Department of Otorhinolaryngology, Head and Neck Surgery, Carl Gustav Carus Faculty of Medicine, TU Dresden, Fetscherstr. 74, 01307 Dresden, Germany

**Keywords:** Anatomy, Evolution, Anthropology, Zoology

## Abstract

The anatomy of the auditory region of fossil hominins may shed light on the emergence of human spoken language. Humans differ from other great apes in several features of the external, middle and inner ear (e.g., short external ear canal, small tympanic membrane, large oval window). However, the functional implications of these differences remain poorly understood as comparative audiometric data from great apes are scarce and conflicting. Here, we measure the sound transfer function of the external and middle ears of humans, chimpanzees and bonobos, using laser-Doppler vibrometry and finite element analysis. This sound transfer function affects auditory thresholds, which relate to speech reception thresholds in humans. Unexpectedly we find that external and middle ears of chimpanzees and bonobos transfer sound better than human ones in the frequency range of spoken language. Our results suggest that auditory thresholds of the last common ancestor of *Homo* and *Pan* were already compatible with speech reception as observed in humans. Therefore, it seems unlikely that the morphological evolution observed in the bony auditory region of fossil hominins was driven by the emergence of spoken language. Instead, the peculiar human configuration may be a by-product of morpho-functional constraints linked to brain expansion.

## Introduction

Humans, chimpanzees (*Pan troglodytes*) and bonobos (*Pan paniscus*) differ in the way they communicate. Frequent use of long-distance loud vocalizations, alongside gestural and short range acoustic signals, characterizes chimpanzees (e.g. pant hoots) and bonobos (e.g. high hoots)^1,2^. Living in distinct fission–fusion societies, such loud call utterances allow them to maintain spatial contact with conspecifics^3–5^, while transmitting information about identity, social status, and physical condition of the caller^6–10^. Humans, on the other hand, typically use spoken language, a unique form of short-distance communication structured around basic sound units called phonemes^11^, although forms of long-distance vocalizations exist (e.g., whistled languages^12^). The ability to combine phonemes to an almost infinite number of meaningful vocal expressions, which gives complexity and plasticity to speech, clearly separates humans from all other primates^13–15^.

Using any form of acoustic communication requires being able to produce, but also to capture specific acoustic signals. Concerning speech, the capacity to capture relevant acoustic information is quantified through two distinct metrics called speech intelligibility and the speech reception threshold^16,17^. Speech intelligibility corresponds to the percentage of speech that a listener can understand, and is mostly related to frequency discrimination of auditory stimuli at the level of the central nervous system and auditory nerve fibres^17,18^. The speech reception threshold, on the other hand, corresponds to the minimum hearing level for speech, and is related to auditory thresholds^16^, which are mainly determined by the functional morphology of the auditory region^19–21^. The external and middle ear collect, transmit and amplify airborne sound pressures that can be characterized through transfer functions that relate airborne sound to middle ear motion or inner-ear sound pressure^19–22^, where these transfer functions determine much of the frequency dependence of hearing function. The inner ear sound sensors determine the absolute sensitivity of the ear to sound^23,24^, and place further limits on the lowest and highest audible sound frequencies^25^.

In this context, it is not surprising that humans and chimpanzees differ in morphological aspects of their external and middle ears. In particular, among hominids, humans have the shortest external ear canal, the smallest tympanic membrane, the largest stapes footplate, the smallest lever length ratio for their malleus/incus complex, and the smallest area ratio between their tympanic membrane and stapes footplate. In contrast, chimpanzees largely fall within great ape variation^26–29^. These morphological differences led some authors to suggest that human audition might have evolved for speech reception^30,31^. This is further supported by the findings that, among primates which have been experimentally tested between 1 and 8 kHz (apart from one specific study^32^), humans show the lowest auditory thresholds on average (i.e., highest hearing sensitivity)^33^. This suggests that the auditory region of fossil hominins, functionally related to the speech reception threshold, could be important for pinpointing the origin of spoken language, especially since other structures involved in vocal communication (e.g., larynx, neural/cerebral tissue) or speech intelligibility (e.g., auditory nerve fibres) are not preserved by fossilisation.

However, empirical evidence for the functional significance of morphological differences between the auditory regions of humans and chimpanzees is dubious. Indeed, while human audition is well studied, and does show low auditory thresholds in the frequency range where phonemes are generally emitted (0.125–8 kHz^34^), great ape audition remains poorly understood, as the only two studies of chimpanzee audition report conflicting results (Elder/Kojima thereafter)^30,32^. Whereas both studies show a typical W-shaped audiogram commonly seen in anthropoids^35^, including relatively low auditory thresholds in high frequencies potentially linked to the use of long-distance vocalizations^36^, they disagree in their comparisons to humans in the frequency range of spoken language. Here, chimpanzees are found to either show higher (Kojima^30^) or lower (Elder^32^) auditory thresholds than humans. These audiometric studies were based on small samples, did not follow standardized protocols and relied on animal training and cooperation^33^. Therefore, robust audiometric data of our closest living relatives are needed to unequivocally assess whether chimpanzees show higher or lower auditory thresholds than humans in the frequency range of speech.

Hence, in this study, (1) we analyse the impact the Elder and Kojima chimpanzee audiograms could have onto the interpretation of the emergence of spoken language, in the phylogenetic context of the evolution of auditory thresholds of extant primates between 1 and 8 kHz, (2) we take a practical, more objective approach to access auditory capacities of 4 chimpanzees, 3 bonobos and 11 humans, by experimentally measuring their middle ear transfer function, using laser-Doppler vibrometry to measure stapes motion, and by analysing their external ear transfer function, via finite-element modelling, which will allow us to directly compare their external/middle ear transfer function (EMTF) between 0.2 and 10 kHz, (3) we assess how chimpanzee/human EMTF magnitude differences compare to published evidence, and determine under which chimpanzee audition model they are more likely to occur, (4) we link our findings to morphological differences between humans, chimpanzees and bonobos, including cochlear dimensions upon which hinges the validity of extending EMTF differences to absolute threshold differences. Finally, we use observations made in points 1–4 to assess whether morphological changes in the auditory region of fossil hominins could be used to track the emergence of spoken language.

## Results

### Evolution of auditory thresholds of extant primates between 1 and 8 kHz

**Figure 1:**
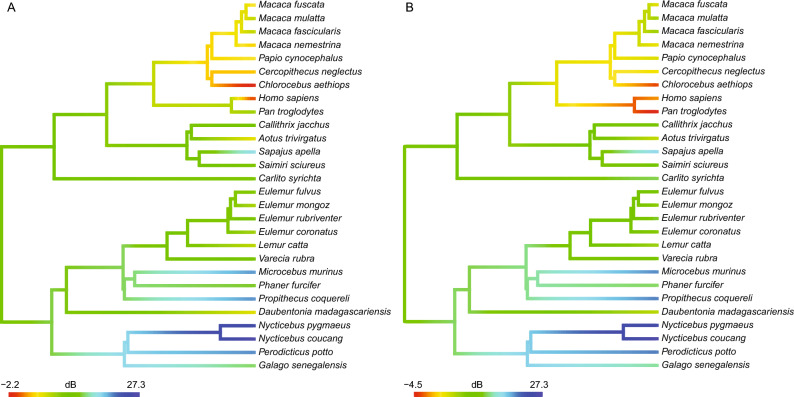
Evolutionary models representing the most likely evolution of the average auditory threshold of primates between 1 and 8 kHz. (**A**) Evolutionary model when considering the Kojima audiogram^30^ for chimpanzees. (**B)** Evolutionary model when considering the Elder audiogram^32^ for chimpanzees. Note the differences at the node of the last common ancestor of *Homo* and *Pan*. (**A**, **B**) The colour scale represents decibels.

Analyses of the average auditory threshold of primates between 1 and 8 kHz (AT18m), using the Kojima audiogram for chimpanzees, suggest that a Brownian evolution model, with a change in the rate of evolution along the branch going from the last common ancestor of *Homo* and *Pan*, and leading to *Homo*, best explains the data (σ1 = 1.0, σ2 = 3.4, AICc = 176.7, Supplementary Fig. 1a). Under this model (Fig. 1a), the ancestral state for the AT18m of the last common ancestor of *Homo* and *Pan* is predicted to be 7.8 dB, to be compared with human AT18m (− 0.1 dB). These results suggest that while evolution of the AT18m was gradual during most of primate history, selection pressures pushed the AT18m to dramatically decrease along the human lineage, after the split between *Homo* and *Pan*.

Alternatively, we find that when using the Elder audiogram for chimpanzees, a Brownian evolution model, with constant evolutionary rate, best explain the data (σ = 1.0, AICc = 175.3, Supplementary Fig. 1b). Under this model (Fig. 1b), the ancestral state for the AT18m of the last common ancestor of *Homo* and *Pan* is predicted to be -1.5 dB. Taken together, these results suggest that the evolution of the AT18m was gradual during primate history, and that the AT18m of humans slightly increased when compared to the ancestral value seen in the last common ancestor of *Homo* and *Pan*.

### The external/middle ear transfer function of humans, chimpanzees and bonobos

**Figure 2:**
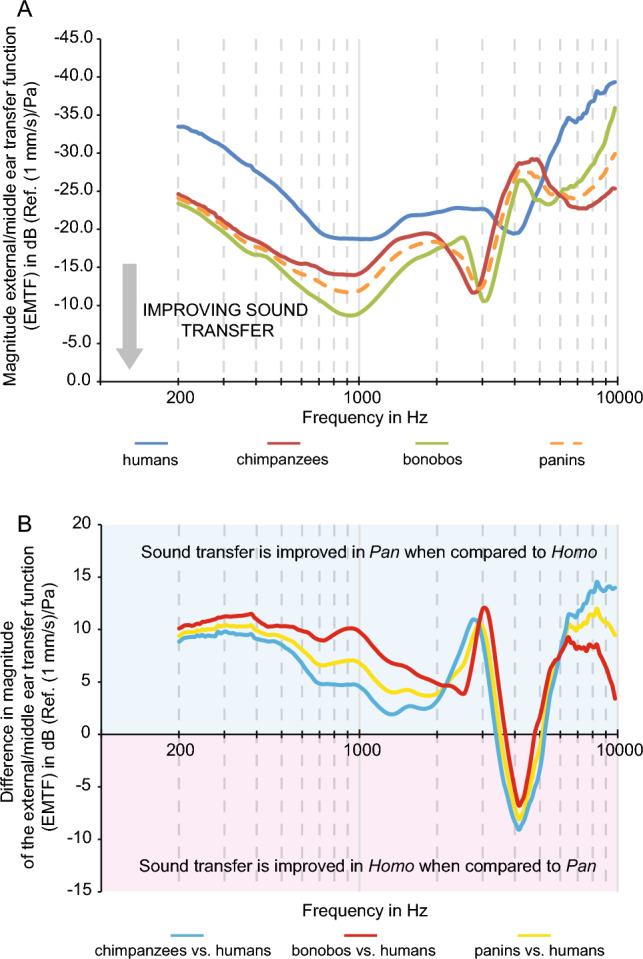
Mean magnitudes of the external/middle ear transfer function (EMTF). (**A**) *H. sapiens* (blue), *P. paniscus* (green), *P. troglodytes* (red) and *Pan* (dashed orange). The y-axis has been reversed to allow comparisons with audiograms in Kojima^30^. (**B**) shows differences in mean magnitudes of the EMTF between humans and *P. paniscus* (red), humans and *P. troglodytes* (blue) and humans and panins (*Pan*; yellow). A similar plot was provided in Elder^32^ to compare audiograms. (**A**, **B**) Note that panins always show higher magnitudes than humans, except between 3.5 and 5.1 kHz.

The external/middle ear transfer function (EMTF) is shaped by the morphology of external and middle ear structures and affects the frequency dependence of auditory thresholds across species^20,21^. It combines the middle ear transfer function (METF) of each species (Supplementary Figs. S2 and S3, Supplementary Tables S1 and S2, Supplementary Text 2), experimentally measured on unfixed cadavers via laser-Doppler vibrometry (Supplementary Text 1), with the pressure gain function of their respective external ear canal (Supplementary Fig. S4; Supplementary Tables S3 and S4), modelled using finite element analysis. Average magnitudes of the EMTF of humans, chimpanzees and bonobos were plotted against sound frequency from 0.2 to 10 kHz (Fig. 2, Supplementary Table S5). As no significant differences were found between magnitudes, peak frequencies and growth slopes of chimpanzees and bonobos across this frequency range (Supplementary Table S6), comparisons will mainly focus on panins (chimpanzees and bonobos) and humans (Table 1).

**Table 1:** Summary statistics and comparison of key characteristics of the external/middle ear transfer function (EMTF) of humans (*Homo*) and panins (*Pan*).

	Humans (*Homo*)	Panins (*Pan*)	*p*_humans vs. panins_
Frequency First Maximum	1.1 [0.6, 1.3]	0.9 [0.6, 1.3]	0.678
Magnitude First Maximum	− 18.7 [− 24.5, − 7.8]	− 11.7 [− 20.6, − 1.3]	0.081
Frequency First Minimum	2.4 [1.3, 3.9]	1.9 [1.3, 3.1]	0.494
Magnitude First Minimum	− 22.8 [− 35.2, − 15.4]	− 18.4 [− 29.5, − 10.1]	0.096
Frequency Second Maximum	**4.0 [3.5, 4.5]**	**2.9 [2.6, 3.2]**	** < 0.001**
Magnitude Second Maximum	− **19.4 [**− **32.4, **− **5.6]**	− **12.2 [**− **17.1, **− **5.0]**	**0.038**
Frequency Second Minimum	–	4.3 [3.7, 5.6]	–
Magnitude Second Minimum	–	− 27.7 [− 37.7, − 22.6]	–
Frequency Third Maximum	–	6.7 [4.8, 9.0]	–
Magnitude Third Maximum	–	− 24.0 [− 38.6, − 5.0]	–

Frequency (in kHz) and magnitude (in dB). Given values are the average and in brackets the minimum and maximum values.

*P* values below 0.05 indicate significant differences (bold).

The average human EMTF shows two maxima (at 1.1 kHz and 4.0 kHz), separated by a minimum (at 2.4 kHz), while the average panin EMTF shows three maxima (at 0.9, 2.9 and 6.7 kHz), separated by two minima (at 1.9 and 4.3 kHz). The magnitude of the average EMTF of panins is generally higher than that of humans (+ 6.5 dB averaged over the studied frequency range), except for a small range between 3.5 and 5.1 kHz (− 4.4 dB) (Fig. 2, Table 2).

**Table 2:** Comparison between humans and panins over frequency range of significantly different magnitudes.

Frequency range	Average difference in magnitude	*p* values
0.200–1.060	-8.8	0.0001–0.048
2.613–3.144	-9.3	0.001–0.038
4.150	8.1	0.038

Frequency in kHz, magnitude in dB. Positive difference in magnitude suggests better hearing sensitivity in humans.

Range of *p* values correspond to lowest and highest *p* value in the considered frequency range.

Statistically, the frequency and magnitude of the first maximum and minimum of the human and panin EMTF does not differ significantly (Table 1, Supplementary Table S6), but the frequency and magnitude of the second maximum are significantly different (*p*_Frequency_ = 4.92 10^–6^, *p*_Magnitude_ = 3.80 10^–2^). Compared to humans, magnitude of the panin EMTF is significantly higher between 0.2–1.1 kHz (+ 8.8 dB) and 2.6–3.1 kHz (+ 9.3 dB) (Table 2, Supplementary Table S6). Conversely, humans only show a significantly higher EMTF magnitude at 4.2 kHz (+ 8.1 dB, *p* = 0.038). Interestingly, chimpanzees show additional areas of higher EMTF magnitude between 6.4 and 7.8 kHz (Supplementary Table S6). The significantly higher EMTF magnitude of panins represents 48.5% of the studied frequency range (logged), whereas the significantly higher EMTF magnitude of humans represents only 0.6% of the same range. Growth of the EMTF between homologous maxima and minima of humans and panins is not significantly different (Supplementary Table S6).

### Statistical comparisons of published chimpanzee/human auditory threshold differences with EMTF results

Linear regression models show that chimpanzee/human differences in auditory thresholds reported by Elder^32^ (Δ_ELDER_) are significantly correlated to the ones reported by Kojima^30^ (Δ_ELDER_ ~ Δ_KOJIMA_, adjusted *p* value = 0.04), with a slope of 0.33, an intercept of − 11.0 dB and a coefficient of determination (adjusted R^2^) of 0.59. Similarly, chimpanzee/human EMTF magnitude differences reported in this study (Δ_EMTF_) are significantly correlated to published chimpanzee/human differences in sound power transmission predicted from circuit models^26^ (adjusted *p* value < 0.001), with a slope of 0.89, an intercept of − 6.1 dB and a coefficient of determination of 0.81.

Comparing EMTF data to published chimpanzee audiograms, we find that Δ_EMTF_ is significantly correlated to Δ_ELDER_ (Δ_EMTF_ ~ Δ_ELDER,_ adjusted *p* value = 0.04), with an AICc of 44.8, a slope of 0.99, an intercept of 2.8 dB and a coefficient of determination of 0.59. In contrast, Δ_EMTF_ is neither significantly correlated to Δ_KOJIMA_ (Δ_EMTF_ ~ Δ_KOJIMA_, adjusted *p* value = 0.11), nor to the average of chimpanzee/human auditory threshold differences reported by Elder and Kojima (Δ_EMTF_ ~ Δ_AVERAGE_, adjusted *p* value = 0.08). These models respectively present AICcs of 48.4 and 47.0, slopes of 0.33 and 0.54, intercepts of − 8.0 dB and − 5.3 dB, and coefficients of determination of 0.31 and 0.43.

The results we obtained for Δ_EMTF_ are best explained by the Δ_ELDER_ model. In comparison, the Δ_KOJIMA_ and Δ_AVERAGE_ models are respectively 6.3 and 3.1 times less probable than the Δ_ELDER_ model to explain our data.

### Morphology of the auditory region of humans and panins

To relate sound transmission to morphology, relevant anatomical structures of the external, middle and inner ears were measured (Supplementary Table S7, S8).

**Figure 3:**
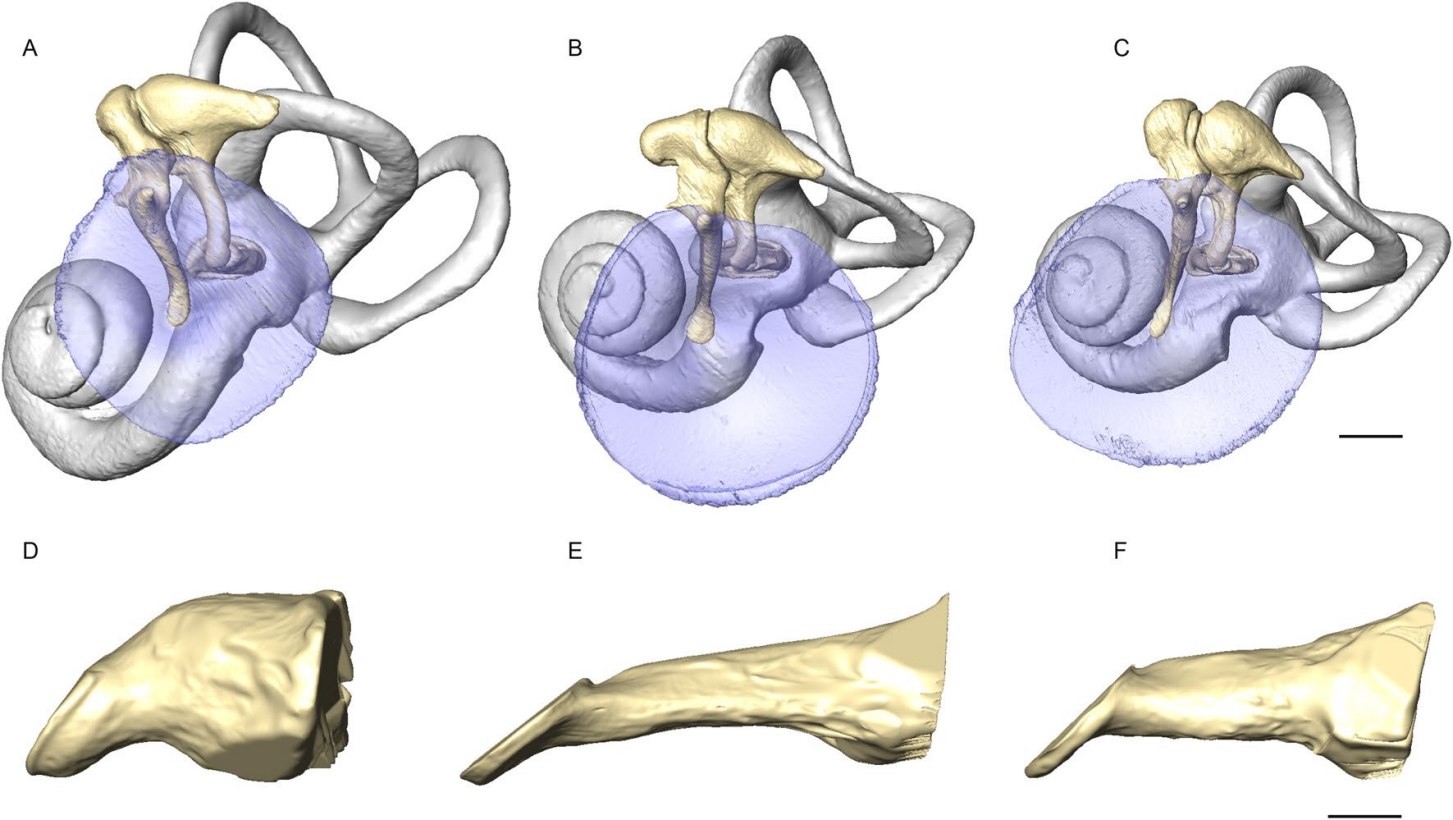
Three-dimensional reconstructions of the in-situ morphology (tympanic membrane [blue], ossicluar chain incl. stapes footplate [bone color]) of the middle ear (and bony labyrinth [grey]) shown from lateral perpendicular to the plane of the stapes footplate of a (**A**) human (CEB-130125), (**B**) chimpanzee (CEB-130093) and (**C**) bonobo (CEB-150021). Reconstructions are based on micro-CT data of phosphotungstic acid stained temporal bone samples. Scale bar, 2 mm. Also shown are virtual casts of the bony external ear canal (left side of the skull, seen from anterior, ear openings are on the right and the tympanic sulcus on the left) of (**D**) a human (ULAC-812), (**E**) a chimpanzee (TAI-11792) and (**F**) a bonobo (AMNH-86857). Scale bar, 5 mm. These figures highlight size differences of the external ear canal, tympanic membrane and ear ossicles between the species, e.g. the distinctly shorter but wider external ear canal and smaller tympanic membrane of humans despite their larger body size (Supplementary Table S7).

Concerning the inner ear, all measured dimensions of the cochlea including fluid-filled volumes and cochlea outline length (a proxy for basilar membrane length) are very similar among hominids overall, with orangutans showing a slightly shorter cochlear length than the African hominids. In contrast, differences exist in the dimensions of the external and middle ears of hominids, for which humans generally appear as outliers. While their surface areas for the articular facets of incus and malleus are similar to what is seen in chimpanzees and bonobos, they show the largest stapes footplate area, the longest functional length of the incus, the smallest tympanic membrane area, the heaviest malleus and incus and the smallest functional length of the malleus of hominids (Fig. 3, Supplementary Tables S7, S8). These metrics result in the lowest impedance transformer ratio^20–22,37^ (i.e. an approximation for the pressure increase achieved by the middle ear at frequencies near its resonance) among hominids, including panins (Supplementary Tables S7, S8). Humans also have the shortest external ear canal of all hominids, including whether looking at bony or cartilaginous parts, which leads to the differences in resonance frequencies observed when comparing humans to chimpanzees and bonobos (Supplementary Tables S7, S8), and which distinctively affect maxima and minima of their respective EMTFs. On the other hand, humans have the widest bony ear canal of measured hominids (Supplementary Tables S7, S8), which could have led to differences in pressure gain magnitude, but is actually compensated by soft tissues, as experimentally shown by comparing humans and chimpanzees^30^. Combined, the apparently derived morphology of the middle and external ears of humans reflects their consistently lower EMTF magnitude when compared to panins.

In contrast, panins generally show a plesiomorphic morphology for the external and middle ear, falling in-between values observed for gorillas and orangutans, but possessing a particularly short external ear canal for great apes, as well as the lightest stapes of hominids. Bonobos are special in showing the smallest stapes footplate area of all hominids, while chimpanzees possess the highest lever length ratio (Supplementary Tables S7, S8).

## Discussion

Knowledge about chimpanzee audition is problematic. The only two published chimpanzee audiograms to date^30,32^ differ in their comparison with human audition. While chimpanzee/human differences in auditory thresholds significantly correlate between the two studies (*p* value = 0.04), there is a difference of 11 dB on average between the chimpanzee/human differences they report. The Elder study supports that chimpanzee auditory thresholds are generally lower than human ones^32^. On the contrary, the Kojima study reports chimpanzee thresholds that are generally higher than human ones^30^. Using two^30^ and three^32^ chimpanzees for their measurements, it would be unlikely that these studies actually sampled the extremes of the range of auditory thresholds of chimpanzees. Instead, it is possible that the Kojima study presented methodological shortcomings. First, while the Elder study measured wild caught chimpanzees^32^, the Kojima one used chimpanzees born in captivity^38^. Second, Elder measured auditory thresholds as the faintest tone intensity eliciting a response^32^, while Kojima reported auditory thresholds as the tone intensities leading to a reaction time of 800 ms^30^. This could result in erroneous differences in auditory thresholds between chimpanzees and humans if their reaction times differ for the faintest tone intensities they can hear. Finally, it has been suggested that Kojima may have overestimated the auditory thresholds of chimpanzees because a 6 cm^3^ coupler was used for calibration and may not have been adequate for the large ear of chimpanzees^33^.

As demonstrated in this study, whether Elder or Kojima reported the actual chimpanzee/human differences in auditory thresholds has major implications on the interpretation of the evolution of human auditory capacities, and their link with the emergence of spoken language (Fig. 1). Indeed, if chimpanzee audition corresponds to audiograms reported by Elder^32^, then human auditory thresholds between 1 and 8 kHz likely increased by a small amount when compared to the last common ancestor of *Homo* and *Pan*. In contrast, if auditory thresholds reported by Kojima are more representative of chimpanzee audition, then human auditory thresholds between 1 and 8 kHz distinctly decreased when compared to the last common ancestor of *Homo* and *Pan*, as the result of a significantly increased evolutionary rate, suggesting adaptive pressure potentially linked to spoken language^17^.

In this study, we analyse the external/middle ear transfer function (EMTF) of humans, chimpanzees and bonobos and demonstrate that panins (chimpanzees and bonobos) generally amplify sound through their external and middle ears to higher magnitudes than humans, in the frequency range of spoken language (0.125–8 kHz; Fig. 2A, B). Humans and panins having similar cochlear dimensions, these magnitude differences may extend to inner ear sound pressure. In this context, it is important to note that chimpanzee/human differences in EMTF magnitude are significantly correlated with chimpanzee/human differences in auditory thresholds as reported by Elder^32^ (adjusted *p* value = 0.04), but not with differences as reported by Kojima^30^ (adjusted *p* value = 0.11). Results we obtain for the EMTF differences are best explained if actual auditory thresholds of chimpanzees are the ones reported by Elder^32^ and not the ones reported by Kojima^30^(relative likelihood ratio = 6.3:1). Additionally, the fact that the measured EMTF differences are best explained if actual auditory thresholds of chimpanzees are the ones reported by Elder^32^, and not an average of values reported by Elder and Kojima^30^ (relative likelihood ratio = 3.1:1), suggests that these studies did not sample extremes of the chimpanzee variation in auditory thresholds. In light of these results, it seems reasonable to conclude that chimpanzee audiograms reported by Elder^32^ best represent their actual auditory thresholds. In this context, discrepancies in the chimpanzee/human differences in auditory thresholds reported by Elder^32^ and Kojima^30^ probably stem from methodological issues found in the latter study, as discussed above. It can be argued that EMTF measurements are not enough to reach this conclusion because they do not take morphofunctional parameters of the inner ear and afferent nerve fibres into account (although cochlea impedance actually affects EMTF measurements and is taken into account). In this regard, it should be noted that morphological parameters of the cochlea of humans and chimpanzees are very similar (humans vs chimpanzees: cochlea length: 40.6 vs. 40.8 mm, cochlea volume: 65.9 vs. 66.7 mm^3^, Supplementary Tables S7, S8), suggesting similar macromechanical properties. Importantly, the fact that chimpanzee/human differences measured via the EMTF and reported by Elder^32^ are isometrically related (slope = 0.99) is unlikely to have occurred by chance alone and shows that the frequency dependence of these two measurements is the same. In this context, the small difference of 2.8 dB on average observed between chimpanzee/human differences measured via the EMTF and reported by Elder^32^, may partially reflect the impact of micromechanical properties of the inner ear and neurophysiological differences between humans and chimpanzees.

Our results have important implications because the Kojima audiogram of chimpanzees^30^ has often been used as empirical support for the presumed uniqueness of human auditory thresholds between 1 and 8 kHz (see Fig. 1A), and its putative co-evolution with the emergence of spoken language during hominin evolution^19,26,31,39^. Subsequent studies supporting and building upon these claims generally relied on mathematical modelling of sound power transmission through the external and middle ears, using both skeletal measurements of ear structures and human soft-tissues characteristics as input data. While our approach shares some limitations with these studies (use of simulated external ear canal pressure gain, impact of signal transduction by cochlear hair cells not considered, increased noise in data at higher frequencies), it greatly improves over them by being based on experimental data accounting by essence for soft-tissue differences between species. Chimpanzee/human EMTF magnitude differences are significantly correlated to chimpanzee/human sound power transmission differences obtained with mathematical models^26,31^ from 0.5 to 5 kHz (adjusted *p* value < 0.001). However, while mathematical models support sound power transmission to be lower in chimpanzees than humans from 1.4 kHz to at least 5 kHz, with a clear decrease in chimpanzees from 3 kHz^19,26,31^, we empirically find that the EMTF of chimpanzees and bonobos actually reaches magnitudes that are similar or higher to that of humans for 99.4% of the frequency range of spoken language, consistent with Elder^32^. Chimpanzee/human magnitude differences diverge by 6.1 dB on average between EMTF measurements and mathematical models^26,31^. These differences likely stem from the fact that mathematical models used human values for the mass and structural properties of the tympanic membrane, mallear attachment, and structural properties of the annular ligament of chimpanzees. All these parameters are known to have a high impact on the output of mathematical models^26,31^, and their native chimpanzee values are part of EMTF measurements.

In contrast to humans, chimpanzees and bonobos are restricted to African tropical forests, even if some populations exploit more open spaces^40^. The low hearing thresholds found in chimpanzees^32^ and inferred for bonobos, in particular to low frequencies, likely reflects a retained catarrhine adaptation^33^ to improve long distance communication within these forest habitats. Every environment is acoustically defined by physical characteristics, which affect sound transmission and ambient noise levels (see ref.^41^). In that regard, dense forests are considered cluttered habitats where acoustic signals generally degrade rapidly with distance^42^. Sound attenuation and background noise levels are however less pronounced at low frequencies^43^, allowing forest animals, including anthropoid primates, to use this frequency range to transmit information over long distances^41,44^. Chimpanzees and bonobos are no exception, and long distance calls they rely on to locate conspecifics do fall in this low frequency range^8,45^. Long distance calls of panins also show substantial acoustic energy around 6–8 kHz^10,46^, fitting with the third maximum observed on their average EMTF (6.7 kHz, Table 1, Fig. 2A), and the second minimum observed in the auditory thresholds of chimpanzees reported in Elder (8 kHz^32^). In dense tropical forests, background noise levels increase above 1 kHz, peak between 2 and 4 kHz and level-off at about 6 kHz, setting de facto an upper limit to low-frequency communication^43,47^. This third maximum (or second minimum^32^), which is not present on the average human EMTF or audiogram, may represent an adaptation of panins to further optimize long distance communication in forest habitat and improve sound localization^36^. Future studies comparing other primate species living in forests versus open habitats, or primate species giving territorial calls versus species which do not, will further help understanding selective constraints put onto the primate auditory system.

When compared to panins, humans likely show a lower auditory threshold (i.e. improved sensitivity) around 4.2 kHz, supported by EMTF data (+ 8.1 dB, *p* value = 0.038) and Elder chimpanzee audiograms^32^ (+ 1.3 dB, 4096 Hz). Voiceless consonants /f/, /s/ and /th/, sometimes considered characteristic features of spoken language^19,39^, occur around these frequencies^34^. While it could be tempting to interpret this result as indicating a selective decrease of the speech reception threshold at these frequencies relevant to spoken language, this human specificity likely has no adaptive value. Indeed, the higher auditory thresholds inferred for panins, in this frequency range, would actually be considered normal, unimpaired hearing in the context of human audiology^48^, and does not prevent them to hear corresponding phonemes. In fact, even auditory thresholds increased by up to 13 dB, defined as a slight hearing loss, would not significantly impact language perception and production, as seen in children^49^. Additionally, it should be noted that voiceless consonants show similarities with voiceless calls of great apes and likely appeared before the split of humans and panins^50,51^, while derived labiodental phonemes like /f/ started to be used after the first divergences of present human populations, and are thus not a defining feature of human spoken language^52,53^. Contrary to what was commonly thought, auditory thresholds reported by Elder for chimpanzees^32^, which are supported by our results, suggest that the speech reception threshold characterizing human hearing, in frequencies relevant to spoken language, did not develop during hominin evolution. Instead, low auditory thresholds were most likely already present in the last common ancestor of *Pan* and *Homo* (Fig. 1b). This outcome casts doubts on the ability to pinpoint the emergence of spoken language from fossilised ear structures of hominins. Indeed, such remains could only ever inform about the auditory thresholds of extinct individuals, which were likely already compatible with speech reception thresholds at the beginning of the hominin lineage. Similar conclusions were drawn for other morphological proxies (e.g., hypoglossal canal size^54^), suggesting that analyses of genes related to human-specific neural mechanisms that control speech production or speech intelligibility could be key to solving this conundrum^15^.

It can be surprising that auditory thresholds of hominins were already compatible with speech reception thresholds before the human-chimpanzee split, well before the emergence of *Homo*, as humans possess a unique combination of derived traits impacting their auditory thresholds^55^. These include the shortest external ear canal, the smallest tympanic membrane, the heaviest incus and malleus, the longest functional length of the incus, the shortest functional length of the malleus, and the largest stapes footplate, among hominids (Fig. 3, Supplementary Tables S7 and S8, Supplementary Figs. S5 and S6). When compared to panins, the small tympanic membrane and lever length ratio of humans likely account for their higher auditory thresholds in the low-frequencies, the short external ear canal account for their lower auditory thresholds at around 4 kHz (Supplementary Table S4), while their large stapes footplate and heavy incus and malleus are likely responsible for the increase in auditory thresholds in the high-frequencies^20,22,56–59^. The specific morphology of the human auditory region was likely primarily impacted by the evolution of the cranial base, which contains the tympanic bone^60^. While the cranial base expanded laterally during hominin evolution, in the context of brain expansion and the shift to bipedalism, the length of the tympanic bone decreased^60^ and the length of the middle ear cavity increased^28^. The tympanic ring, the manubrium of the malleus and the external ear canal, co-varying structures developmentally integrated with the tympanic bone^61^, were directly affected by these changes and became smaller, while the functional length of the incus, bridging the middle ear cavity, became longer^28^. Brain expansion also led to increase the interaural distance, which correlate to lower high-frequency cut-off^35^, likely explaining increases in incus and malleus masses and stapes footplate area.

Consequently, it appears that the peculiar human ear likely emerged as a by-product of the evolution of the human cranial base through brain expansion. Overall, these morphological changes resulted in higher auditory thresholds in humans when compared to the last common ancestor of *Homo* and *Pan*, though still one of the lowest auditory thresholds among primates between 1 and 8 kHz. Spoken language likely evolved in this context, the speech reception threshold matching constrained human auditory thresholds, not the contrary. As a result, the evolution of the auditory region of fossil hominins may rather reflect the evolution of brain expansion, and be of little information about the origin of language.

## Materials and methods

### Models for the evolution of the average auditory threshold between 1 and 8 kHz in primates

To analyse the evolution of the average auditory threshold of primates between 1 and 8 kHz (AT18m), we first compiled primate audiograms from the literature (Supplementary Table S9). The range between 1 and 8 kHz was chosen because spoken language occurs in this range and all published audiograms contain it. The dataset we used was composed of 13 behavioural audiograms using speakers, 4 behavioural audiograms using headphones and 11 audiograms obtained from measuring auditory brainstem responses (ABR) in sedated specimens. When obtained from the same species, ABR, headphone-based and speaker-based behavioural audiograms show similar patterns, but differ in average auditory thresholds^33,62^. Because our dataset mostly consists of speaker-based behavioural audiograms, we had to correct auditory thresholds of ABR and headphone-based audiograms to allow comparisons. To do so, we first computed correction factors as threshold differences between ABR and speaker-based behavioural audiograms of *Lemur catta* (Supplementary Table S9) and *Nycticebus coucang* (Supplementary Table S9), and between headphone-based and speaker-based behavioural audiograms of *Macaca fuscata* (Supplementary Table S9) and *Macaca fascicularis* (Supplementary Table S9), at 11 different frequencies between 1 and 8 kHz. Then, for each tested frequency, we computed the average between correction factors of *Lemur catta* and *Nycticebus coucang*, and between correction factors of *Macaca fuscata* and *Macaca fascicularis,* and used these average correction factors to respectively scale auditory thresholds of ABR and headphone-based audiograms to auditory threshold levels of speaker-based behavioural audiograms. Note that while this correction is tentative, because only based on two species in each case, the average difference we observe between correction factors of *Lemur catta* and *Nycticebus coucang* (3.0 [1.2–6.5] dB), and between *Macaca fuscata* and *Macaca fascicularis* (1.7 [0.0–4.6] dB), respectively remain much lower than the average difference observed between ABR and behavioural audiograms (15.8 [8.1–26.1] dB), and lower than the average difference between headphone-based and speaker-based audiograms (5.0 [0.0–10.0] dB). This suggests that incorporating uncorrected ABR and headphone-based audiograms in our analyses would likely have led to higher error levels than using the imperfect correction factors proposed here. We used speaker-based and corrected audiograms to compute the AT18m of primates’ species. To do so, we computed the integral of each audiogram between log_10_(1) and log_10_(8) and divided the result by (log_10_(8) − log_10_(1)). The primate AT18ms were then used in R 4.2.0 (R Foundation for Statistical Computing, Vienna, Austria), along with a time-calibrated phylogenetic tree, to assess the likelihood of various evolutionary models, using packages motmot 2.1.3^63^, phytools 1.2.0^64^ and Geiger 2.0.10^65^. The phylogeny we used follows published cladograms^66,67^ and divergence dates were obtained from TimeTree^68^. Branching was modified when divergence dates were in conflict with published phylogenies. For each assumption on the chimpanzee AT18m (Elder or Kojima) we tested 10 different evolutionary scenarios: Brownian motion with 0 to 4 rate shifts, Pagel’s λ, Pagel’s δ, Pagel’s κ, Ornstein–Uhlenbeck and accelerating/decelerating rates (ACDC). These scenarios were compared using their AICc and the evolutionary tree corresponding to the best one was selected for each assumption (Elder or Kojima). These two evolutionary trees were then used with their respective chimpanzee AT18m and the AT18ms of other primate species to infer ancestral values of the AT18m at each node, using the function fastAnc() from the package phytools 1.2.0^64^.

### Experimental investigations of the METF

All methods were carried out in agreement with relevant guidelines and regulations. The experimental protocols were approved by an institutional committee (EK59022014, Technische Universität (TU) Dresden, Ethikkommision an der TU Dresden, Fetscherstr. 74, 01307 Dresden, Germany). Informed consent was obtained from all subjects and/or their legal guardian(s). Investigations were performed on unfixed, defrosted cadaveric specimens. These conditions give results similar to living ears in humans^22,69^. Twelve human temporal bones (from 11 donors) were included in the study, as well as 8 ears for *Pan troglodytes* (4 individuals) and 5 ears for *Pan paniscus* (3 individuals).

Preparation and setup followed published protocols^70,71^ (for details see Supplementary Text 1). A mastoid approach and a posterior tympanotomy were performed to gain access to the middle ear. Stapes footplate velocity was measured in response to sound stimulation at the tympanic membrane. Sound was stimulated via an insert earphone in the ear canal and measured with a probe microphone in front of the tympanic membrane. Velocity of the footplate was measured with a laser Doppler Vibrometer (LDV) via the middle ear access (Supplementary Fig. S7). For morphological reasons, we could not measure the velocity of the stapes footplate along its piston-like axis of motion. We estimate that the angle between the laser beam and the motion axis (30–50°) results in a bias of 1–4 dB for all measurements.

Excitation was done with a multi-sinus signal at 0.1–10 kHz, with a resolution of about 50 Hz, and a sound pressure of approximately 94 dB SPL. The middle ear transfer function (METF) was calculated as stapes footplate velocity divided by the sound pressure in front of the tympanic membrane. It was determined in the form of a complex frequency response function averaged from 20 measurement frames. In some cases, the frequency response had to be concatenated from consecutive measurements over different overlapping frequency ranges. METFs of different specimens were resampled to a common logarithmic frequency scale and converted to decibels, with 1 mm s^−1^/Pa as reference, before averaging.

Since the volume of the tympanic cavity and surrounding spaces affects the METF, particularly in the low frequencies^72,73^, opening the middle ear cavity likely affected reported values. However, since chimpanzee and humans share similar middle ear volumes^26^, interspecific comparisons remain meaningful.

### Modelling pressure gain in the external ear canal (EEC)

Simulations were performed using finite element analysis of a human model^74,75^ (see Supplementary Text 1) composed of the external ear canal (bony plus cartilaginous parts), the full middle ear (including joints and ligaments/tendons) and a simplified model of the cochlea based on^76^ (Supplementary Fig. S8). The middle ear part served as a realistic terminating impedance to calculate the pressure gain in the EEC. Model parameters (mechanical properties, length and diameter of the ligaments and joints) are listed in the Supplementary Table S10. Geometry and parameters of the EEC model were adapted such that its pressure gain transfer function matches average experimental data from literature^76,77^.

The EEC was subsequently scaled to chimpanzee and bonobo dimensions to get simulation data for all three species. The middle ear morphology was not altered. Following (21), in which the pressure gain of a chimpanzee ear canal was shown to have magnitude comparable to the human subjects, EEC wall impedance of bonobos and chimpanzees was adapted to match the magnitude of the pressure gain of the human EEC model. The pressure gain was calculated between 0.2 and 7 kHz (humans), or 0.2–5 kHz (panins), as the ratio between a pressure of 1 Pa applied at the entrance of the EEC and the pressure obtained in front of the tympanic membrane. Since the model has only been validated up to the first resonance, calculations were stopped before the second resonance.

### Statistical comparisons of the EMTFs of humans and panins

Statistical differences between magnitudes of the EMTF of humans (n = 11), chimpanzees (n = 4), bonobos (n = 3) and panins (n = 7) were tested between 0.2 and 9.8 kHz, by comparing magnitudes every 0.03 octaves. In addition, we tested for statistical differences between frequencies and magnitudes of the first, second and third maxima, as well as for the first and second minima. We also tested for statistical differences between growth rates of the EMTF between 0.2 and 9.8 kHz (slopes 1–6). Statistical analyses were done in R 4.0.3 (R Foundation for Statistical Computing, Vienna, Austria). For all tests, we first used a F-test to compare variances between groups of interest. We then used a t-test to compare group means, or a Welch t-test if variances statistically differed. Since we did a large number of statistical comparisons, we controlled for the false discovery rate by using the function “p.adjust” of R, with the method “fdr”. Complete statistical analyses are provided in Supplementary S6 and includes means, *p* values and F-values.

### Statistical comparisons of published chimpanzee/human magnitude differences and EMTF results

To compare chimpanzee/human EMTF magnitude differences (Δ_EMTF_) to published chimpanzee/human auditory threshold differences (Δ_ELDER_, Δ_KOJIMA_)^30,32^, and published chimpanzee/human sound power transmission differences^26^, we first subtracted human values from chimpanzee values (in the case of audiograms) or chimpanzee values from human values (in the case of EMTF and sound power transmission), for all relevant measured frequencies (Δ_ELDER_, Δ_KOJIMA_: 125, 250, 500, 1000, 2000, 4000, 8000 Hz; sound power transmission: 125, 250, 500, 1000, 1500, 2000, 2500, 3000, 3500, 4000, 4500, 5000 Hz; Δ_EMTF_: 125, 250, 500, 1000, 1500, 2000, 2500, 3000, 3500, 4000, 4500, 5000, 8000 Hz). Measuring differences that way allows negative values at a given frequency to indicate increased hearing sensitivity in chimpanzee when compared to human, as depicted in Δ_ELDER_^32^. An average set (Δ_AVERAGE_) was also computed by averaging Δ_ELDER_ and Δ_KOJIMA_. To best compare with Δ_ELDER_ and Δ_KOJIMA_, the value of Δ_EMTF_ at 125 Hz was extrapolated by 1) fitting 2nd degree polynomial regressions to EMTF data of chimpanzees and humans between 200 and 400 Hz (31 frequencies sampled, R^2^_Chimpanzee_ = 1, R^2^_Human_ = 1) and between 200 and 800 Hz (61 frequencies sampled, R^2^_Chimpanzee_ = 1, R^2^_Human_ = 1), 2) using these polynomial regressions to predict the EMTF values of chimpanzees and humans at 125 Hz, 3) averaging the two predictions for chimpanzees and humans and 4) computing the chimpanzee human differences as described above, using the mean predicted EMTF values. The value of Δ_EMTF_ at 125 Hz was independently verified using the relationship between Δ_EMTF_ and chimpanzee/humans sound power transmission differences (excluding 125 Hz values; − 6.0 dB vs. − 5.9 dB). These datasets were used in R to assess linear correlations between Δ_ELDER_ and Δ_KOJIMA_, and between Δ_EMTF_ on the one hand, and Δ_ELDER_, Δ_KOJIMA,_ Δ_AVERAGE_ and sound power transmission differences on the other hand. *P* values, slopes, intercepts, adjusted R^2^ and AICc were obtained from these regression models, when relevant. AICc were used to compare likelihoods of chimpanzee/human auditory threshold differences in the context of measured Δ_EMTF._ We controlled for the false discovery rate by using the function “p.adjust” of R, with the method “fdr”.

### Morphological investigation of the auditory region

Temporal bones and ossicles of modern humans, chimpanzees and bonobos were scanned with the micro-CTs BIR ACTIS 225/300 or Bruker^TM^SkyScan 1173, or with the X-ray Nanotomograph Bruker^TM^SkyScan 2211. A list of scanned specimens is provided in Supplementary Table S8, with details on image resolution and available morphological structures. Three-dimensional surface models of external ear canals, temporal bones and ear ossicles were done in Avizo 7.1–9.4 (Visualization Science Group; Burlington, MA, USA), using the Segmentation editor, or the Isosurface module for isolated ossicles. Right ear structures were segmented, or left ones were mirrored.

Cochlea length was measured in R as the sum of the distances between each successive landmark placed along the external wall of the cochlea, from above the round window to the apex of the cochlea. Landmarking was done in Avizo. Cochlea volume consists of the addition of the volumes of the perilymphatic and endolymphatic spaces of the cochlea. These spaces were segmented in Avizo on contrast-enhanced soft-tissue specimens^78^ and their volumes were calculated using the same software.

Measurements of areas enclosed by the tympanic sulcus and the oval window, as well as ossicle functional lengths, follow protocols presented in^28^. Landmarking was done in Avizo.

Surface areas of ossicle articular facets were measured in Geomagic Studio20 (Raindrop Geomagic Inc, Morrisville, NC, USA) by delineating the articular facets on the 3D surface models and using the ‘compute surface area’ module.

Lengths and average diameters of bony ear canals were measured in Avizo, for four specimens per species (one side only). Lengths were taken along the central trajectories of 3D surface models of bony ear canals, from the projection of the lateral-most point of the tympanic membrane to the projection of porion. Dimensions of the cartilaginous EEC of humans, chimpanzees and bonobos were obtained by multiplying the length of the bony EEC by a factor of 1.5. This factor was verified on CT scans of chimpanzees (median 1.54, *n* = 8) from the Digital Morphology Museum of Kyoto University (http://www2.ehub.kyoto-u.ac.jp/databases/printeg_view/printeg.php?db=prict). This factor is also found for humans^55^ and using it, we obtain human EEC lengths that fall into normal variation^79^. Cross-sectional areas of bony ear canals were computed at 50% of their lengths, using a custom-made script and landmarks placed along the cross-section.

Masses of ear ossicles were obtained using a precision balance (± 0.01 mg, Sartorius^TM^ BP 210 D) on isolated ear ossicles of humans (n = M25, I26, S22), chimpanzees (n = M15, I15, S7), bonobos (n = M1, I1, S0), gorillas (n = M1) and orangutans (n = M7, I7, S2). Stapes mass of bonobos was estimated from their average CT volume of 0.99 mm^3^.

### Supplementary Information

Supplementary Information 1.

Supplementary Information 2.

Supplementary Information 3.

Supplementary Information 4.

Supplementary Information 5.

Supplementary Information 6.

Supplementary Information 7.

## Data Availability

All data needed to evaluate the conclusions in the paper are present in the paper and/or the Supplementary Materials. Surface reconstructions and landmarks used for measuring morphological dimensions are available from the corresponding author on reasonable request.
