# Supplementary material for: Auditory thresholds compatible with optimal speech reception likely evolved before the human-chimpanzee split
Source: Sci Rep. 2023 Nov 25;13:20732. doi: 10.1038/s41598-023-47778-2 (PMC10676368; doi:10.1038/s41598-023-47778-2)
Supplement: Supplementary file 1 — Supplementary Information 1. [file 41598_2023_47778_MOESM1_ESM.pdf]

Supplementary Information for

Hearing capacities optimally capturing speech frequencies evolved before the human-chimpanzee split

Authors

Alexander Stoessel[1,2,5 *], Romain David[3,5*], Matthias Bornitz[4], Steffen Ossmann[4], Marcus Neudert[4*]

Affiliations

[1] Institute of Zoology and Evolutionary Research, Friedrich Schiller University Jena, 07743 Jena, Germany.

[2] Max Planck Institute for Evolutionary Anthropology, 04103 Leipzig, Germany.

[3] Centre for Human Evolution Research, Natural History Museum, London SW7 5BD, UK.

[4] Department of Otorhinolaryngology, Head and Neck Surgery, Carl Gustav Carus Faculty of Medicine, TU Dresden, 01307 Dresden, Germany.

[5] These authors contributed equally: Alexander Stoessel and Romain David.

Supplementary Text 1

Experimental investigations of the METF

Sample. Investigations were performed on intact, unfixed, defrosted cadaveric specimen. Under these conditions the acoustic properties have been shown to be similar to those in the vital human ear (1, 2). Panin samples were complete head specimens, while human samples were temporal bone specimens. For *Pan troglodytes* 8 ears from 4 individuals were measured and for *Pan paniscus* 5 ears from 3 individuals. One side of individual CEB160008 was already damaged and could not be evaluated. Twelve human temporal bone specimens from 11 donors were included in the study. Preparation and setup were the same for all specimens.

Preparation. The skin was already removed at most samples. In some panin samples the skin and auricle had to be detached from the bone and had to be folded away. After mastoidectomy an extended posterior tympanotomy was performed. Removal of the facial nerve enabled for sufficient access to the tympanic

cavity with exposure of the stapes footplate. The ossicular chain or its ligaments were not damaged, thus retaining the mechanical and dynamical properties of the middle ear. A small square of reflective foil (0.5 mm^2) was placed on the stapes footplate for laser Doppler vibrometer (LDV) measurements. A small hole was drilled into the anterior wall of the ear canal in order to place a probe microphone in front of the tympanic membrane above the umbo. To maintain physiological compliance of the middle ear, the specimens were continuously moistened during the measurements.

Experimental Setup. The complete head specimens were softly placed at the experimental table and the temporal bone specimen were mounted with articulated clamps. An audiometric insert earphone (eartone 3A) was placed into the remaining cartilaginous part of the ear canal. The applied sound pressure was measured with a probe microphone (ER-7c) in front of the tympanic membrane. The velocity of the stapes footplate was measured using a Laser Doppler Vibrometer (LDV) (CLV 700 laser head and CLV 1000 controller unit, Polytec, Waldbronn, Germany); setup see Supplementary Information SI Fig. S6. The laser head was mounted to a micro manipulator and this assembly was then connected to a standard surgical microscope. The laser beam was focused with the micro manipulator to the reflective foil at the center of the stapes footplate. Due to the morphology of the temporal bone it is not possible to measure the velocity of the stapes footplate in the direction of the piston-like axis of stapes motion. The angle of measurement between the Laser beam and the normal direction of the stapes footplate was estimated to lie between 30-50° for all measurements. This corresponds to a bias between 1 to 4 dB in the METF.

Measurements and data preparation. Measurements were done with data acquisition boards (NI PXI4496-input channels, NI PXI6281-output channel, NI PXI1033 chassis) and software based on LabView (both National Instruments, Austin, TX, USA). The excitation signal for the insert earphone was a multi-sinus signal in the frequency range of 0.1 to 10 kHz with a resolution of about 50 Hz, generating a sound pressure of approximately 94 dB SPL.

The middle ear transfer function (METF) was obtained as the complex transfer functions H(jω) in the frequency domain, calculated from two measured signals x(t) and y(t), where x(t) is the reference signal, sound pressure in front of the tympanic membrane and y(t) is the response signal, the stapes footplate velocity. The measured time signals x(t) and y(t) were Fourier transformed to get complex functions of frequency X(jω) and Y(jω). X*(jω) and Y*(jω) are the corresponding conjugate complex functions. Mean auto power density and mean cross power density were calculated from n=20 measurement frames as:

$$S_{xx}(j\omega) = \frac{1}{n}\sum_{i=1}^{n}[X_i(j\omega) * X_i^*(j\omega)],$$

$$S_{yy}(j\omega) = \frac{1}{n}\sum_{i=1}^{n}[Y_i(j\omega) * Y_i^*(j\omega)],$$

$$S_{xy}(j\omega) = \frac{1}{n}\sum_{i=1}^{n}[X_i(j\omega) * Y_i^*(j\omega)],$$

$$S_{yx}(j\omega) = \frac{1}{n}\sum_{i=1}^{n}[Y_i(j\omega) * X_i^*(j\omega)].$$

The transfer function was then calculated as

$$H_3 = \frac{1}{2}\left(\frac{S_{xy}}{S_{xx}} + \frac{S_{yy}}{S_{yx}}\right)$$

Only the magnitude |H| of the transfer function H(jω) is displayed in the diagrams and used in further data preparation.

In some cases, the frequency response had to be concatenated from consecutive measurements over different overlapping frequency ranges.

Before averaging the METFs of different specimens, the respective METFs were resampled to a common logarithmic frequency scale and converted to decibel with 1 mm·s^{-1}/Pa as reference. The means were first calculated per individual in case both ears were measured and subsequently species means were computed.

Since the volume of the tympanic cavity and surrounding spaces have an effect on the METF, particularly in the low frequencies (3, 4), opening the middle ear cavity during temporal bone preparation is expected to have affected recorded METFs. However, since chimpanzee and humans have largely similar middle ear volumes (5), interspecific comparisons should not be hindered by these procedures.

Modelling pressure gain in the external acoustic meatus (EEC)

Model simulations. We used a finite element model of the human middle ear and external acoustic canal (6, 7) for the simulations (SI Fig. S7). The middle ear part of the model served as realistic terminating impedance for the calculations of pressure gain in the EEC. The EEC of the model was subsequently adapted to chimpanzee and bonobo anatomy to get simulation data for all three species.

Model structure and parameters. The model is implemented in ANSYS® (Ansys Inc., Canonsburg, Pennsylvania, USA) and consists of the EEC, the tympanic membrane, the ossicles (malleus, incus and stapes), the incudomallear and incudostapedial joint, ligaments/tendons and a simplified model of the cochlea. The EEC is modelled as an acoustic fluid that can be described by the Helmholtz equation. The geometry of the EEC is a 3D volume adapted from published data (8) and corresponds to an average human ear canal. A surface impedance was added to the outer EEC surface to account for the damping of the canal wall. Parameters of the EEC model were adapted such that its pressure gain transfer function matches the average experimental data from literature (8-10).

The tympanic membrane (TM) is modelled as a three-layer shell with orthotropic material behavior. This represents its histological structure consisting of the epidermal layer, the lamina propria and the mucosal epithelial layer (11). Between the structural elements of the TM and the acoustical elements of the EEC a strong coupling is established.

The ossicles, the joints capsules and the stapedial annular ligament (SAL) are modelled as isotropic elastic bodies. The connection between the tympanic membrane and the malleus handle is modelled by rigid kinematic constraints as is the connection between the ossicles and the joints.

The ligaments and tendons (except SAL) are represented by cylindrical beam elements with isotropic elastic material behavior. Additionally, for all soft tissue components, i.e. tympanic membrane, joints and ligaments and tendons, a structural damping was applied. For the cochlea a simplified mass-spring-damper model was used based on (10). As boundary conditions the circumference of the tympanic membrane is simply supported, i.e. the translational DOF are fixed, and the ends of the ligaments and tendons are clamped. The stapedial annular ligament is simply supported on the outer circumferential surface corresponding to the oval window.

The model parameters (mechanical properties, length and diameter of the ligaments and joints) are listed in the Supplementary Data Table S9.

Model adaption to chimpanzee and bonobo simulations. To estimate the pressure gain of the chimpanzee and the bonobo the length of the EEC and its diameter was scaled according to the data in Table S7. The middle ear morphology was not altered. The damping on the canal wall (ear canal impedance) was adapted for bonobos and chimpanzees to match the magnitude of the pressure gain in the human ear canal model. This assumption was drawn from (12) in which the pressure gain of a chimpanzee ear canal was shown to have magnitude comparable to the human subjects.

Pressure gain was calculated between 0.2 and 7 kHz (humans) or 5 kHz (panins). A pressure magnitude of 1 Pa was applied at the entrance of the external auditory canal and the pressure in front of the middle of the tympanic membrane was calculated. The ratio between the two pressures represents the EEC pressure gain.

As the model has been validated only up to the first resonance of the EEC the calculations were terminated before the second EEC resonance.

Supplementary Text 2

Characterization/description of the human and panin middle ear transfer function (METF)

Average magnitudes of the METF of humans, chimpanzees and bonobos were plotted against sound frequency from 0.2 to 10 kHz (SI Figs. S1 and S2, Tables S1 and S2). To test for statistical significance between means of peak magnitude (first maximum), frequency of the peak magnitude and magnitude of the METF along its entire progression, Student's t-tests were used (after testing for equal variances) utilizing the Independent Two-Sample T-Test Calculator provided at https://www.icalcu.com/. In the case of unequal variance, the Welch's test was chosen. Data points for means of peak magnitude and frequency of the peak magnitude of the METF were collected by choosing the peak magnitude of every single individual (average

of left and right ear, if present). To test statistical significance of differences of means of the METF along its entire progression 15 frequencies describing the entire progression (comprising 5.5 octaves, based on frequencies often depicted in audiograms) were compared. Here, individuals (average of left and right ear, if present) were analyzed.

Up to approximately 4 kHz the overall pattern is similar between the three species: the METF increases up to a peak at approximately 1 kHz, with a slope between 0.2-0.7 kHz of 7 dB/octave in humans and 6 dB/octave in chimpanzees and bonobos, then subsequently decreases until 4 kHz with a slope of -9 dB/octave in bonobos and -8 dB/octave in humans and chimpanzees. From 4 kHz onwards, the METF of humans continues its decrease, whereas a second peak in METF appears at 7.2 kHz (max. -24 dB) in chimpanzees and at 5.4 kHz (max. -24 dB) in bonobos. The small peak around 3 kHz arises because of the averaging process and the very different METFs. The shift in pattern of the METF after 4 kHz results in distinct differences in magnitude between panins and humans (up to 15 dB) in the high frequencies of the measuring range.

The peak magnitude of the METF, around 1 kHz, shows significant differences between humans, chimpanzees and bonobos (SI Figs. S1, S2, SI Tables S1 and S2). The magnitude of the peak is significantly smaller ($p=0.0282$) in humans (-17 dB, ± 4.2 dB) than in chimpanzees and bonobos combined (panins) (-12 dB, ± 4.5 dB) and slightly higher in bonobos (-10 dB, ± 7.3 dB) than in chimpanzees (-14 dB, ± 1.7 dB), but this is not statistically significant ($p=0.500$). The frequency at which the peak occurs seems slightly lower in the chimpanzee species (0.78 kHz, ± 0.239 kHz) than in humans (0.91 kHz, ± 0.172 kHz), but this is again not statistically significant ($p=0.19073$).

In addition to variations in peak magnitude of the METF, significant differences also exist between humans, chimpanzees and bonobos across the entire measured frequency range, with panins showing significantly higher magnitudes than humans in the low frequencies, between 0.2-1 kHz, and in the upper mid- to high frequencies, between 5 kHz and 9.5 kHz, while magnitudes between 1-5 kHz are not statistically different. In the low frequencies, differences in magnitude increase with decreasing frequency, reaching 10 dB at 0.25 kHz. In the high frequencies, differences are kept high over the frequency range, with magnitudes about 13 dB higher in panins than humans, reflecting the characteristic increase seen in panins above 4 kHz.

1. Puria S, Peake WT, & Rosowski JJ (1997) Sound-pressure measurements in the cochlear vestibule of human-cadaver ears. *J. Acoust. Soc. Am.* 101(5 Pt 1):2754-2770. **29**
2. Rosowski JJ, Davis PJ, Donahue KM, Merchant SN, & Coltrera MD (1990) Cadaver middle ears as models for living ears: comparisons of middle ear input immittance. *Ann. Otol. Rhinol. Laryngol.* 99(5):403-412. **56**
3. Huang GT, Rosowski JJ, & Peake WT (2000) Relating middle-ear acoustic performance to body size in the cat family: measurements and models. *J. Comp. Physiol. A* 186(5):447-465. **59**
4. Voss SE, Rosowski JJ, Merchant SN, & Peake WT (2000) Acoustic responses of the human middle ear. *Hear. Res.* 150(1-2):43-69. **60**
5. R. Quam *et al.*, Early hominin auditory capacities. *Sci Adv* **1**, e1500355 (2015).

6. Bornitz M, Hardtke HJ, & Zahnert T (2010) Evaluation of implantable actuators by means of a middle ear simulation model. *Hear. Res.* 263(1-2):145-151. **61**
7. Oßmann S, Carus G, Bornitz M, Fleischer M, & Zahnert T (2015) On the influence of anatomical variations on METF–theoretical investigations using a finite element model. *Memro 2015, 7th International Symposium on Middle-ear Mechanics in Research and Otology, Aalborg, Denmark*. **62**
8. Stinson MR & Lawton BW (1989) Specification of the geometry of the human ear canal for the prediction of sound-pressure level distribution. *J. Acoust. Soc. Am.* 85(6):2492-2503. **65**
9. Hammersho/i D & Mo/ller H (1996) Sound transmission to and within the human ear canal. *The Journal of the Acoustical Society of America* 100(1):408-427. **63**
10. Hudde H & Engel A (1998) Measuring and modeling basic properties of the human middle ear and ear canal. Part III: Eardrum impedances, transfer functions and model calculations. *Acta Acustica united with Acustica* 84(6):1091-1108. **64**
11. Schmidt S-H & Hellström S (1991) Tympanic-Membrane Structure–New Views. *ORL* 53(1):32-36. **66**
12. Kojima S (1990) Comparison of auditory functions in the chimpanzee and human. *Folia Primatol. (Basel)* 55(2):62-72. **21**

.

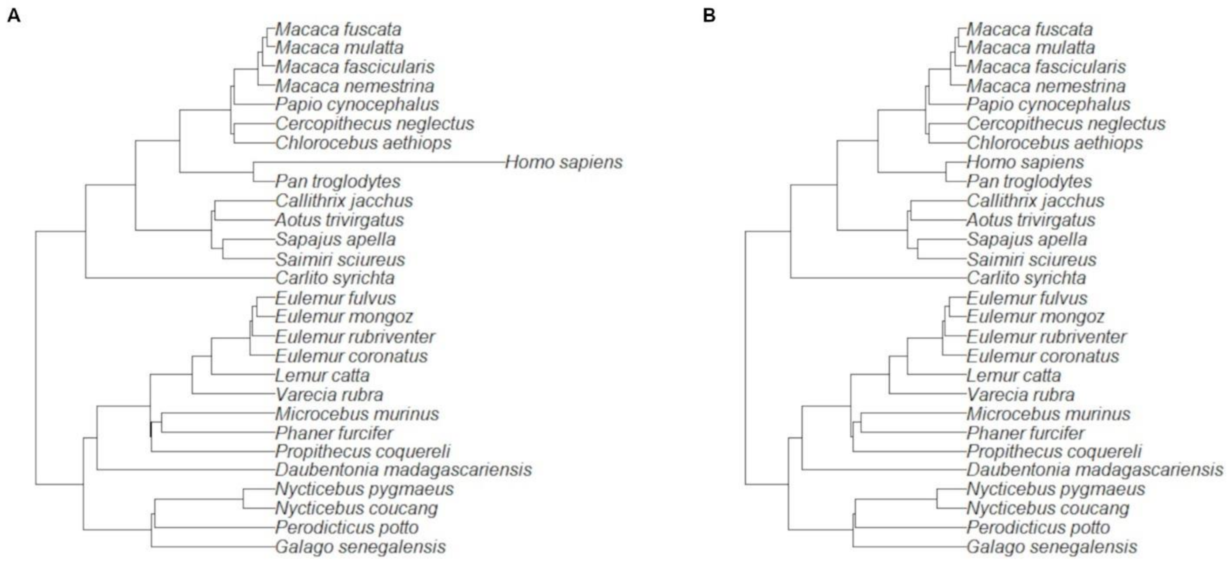

Supplementary Fig. S1. Trees reflecting the best models for the evolution of the average auditory threshold between 1-8 kHz in primates. (A-B) Branch length reflects divergence time, multiplied by evolutionary rate along the branch. (A) Best evolutionary model when the Kojima chimpanzee audiogram is considered. (B) Best evolutionary model when the Elder chimpanzee audiogram is considered.

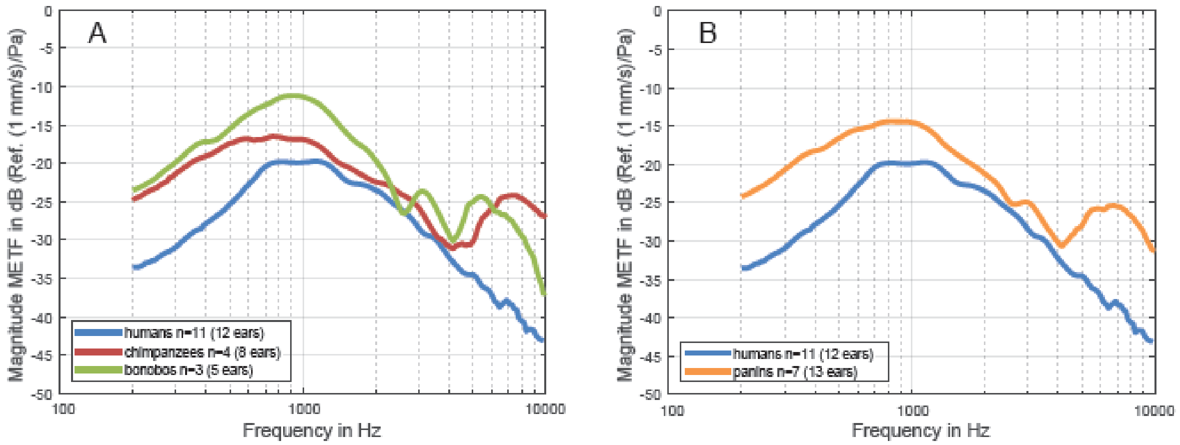

Supplementary Fig. S2. Average magnitude of middle ear transfer function. (A) *H. sapiens* (blue), *P. paniscus* (green) and *P. troglodytes* (red) and (B) of humans (blue) and panins (orange).

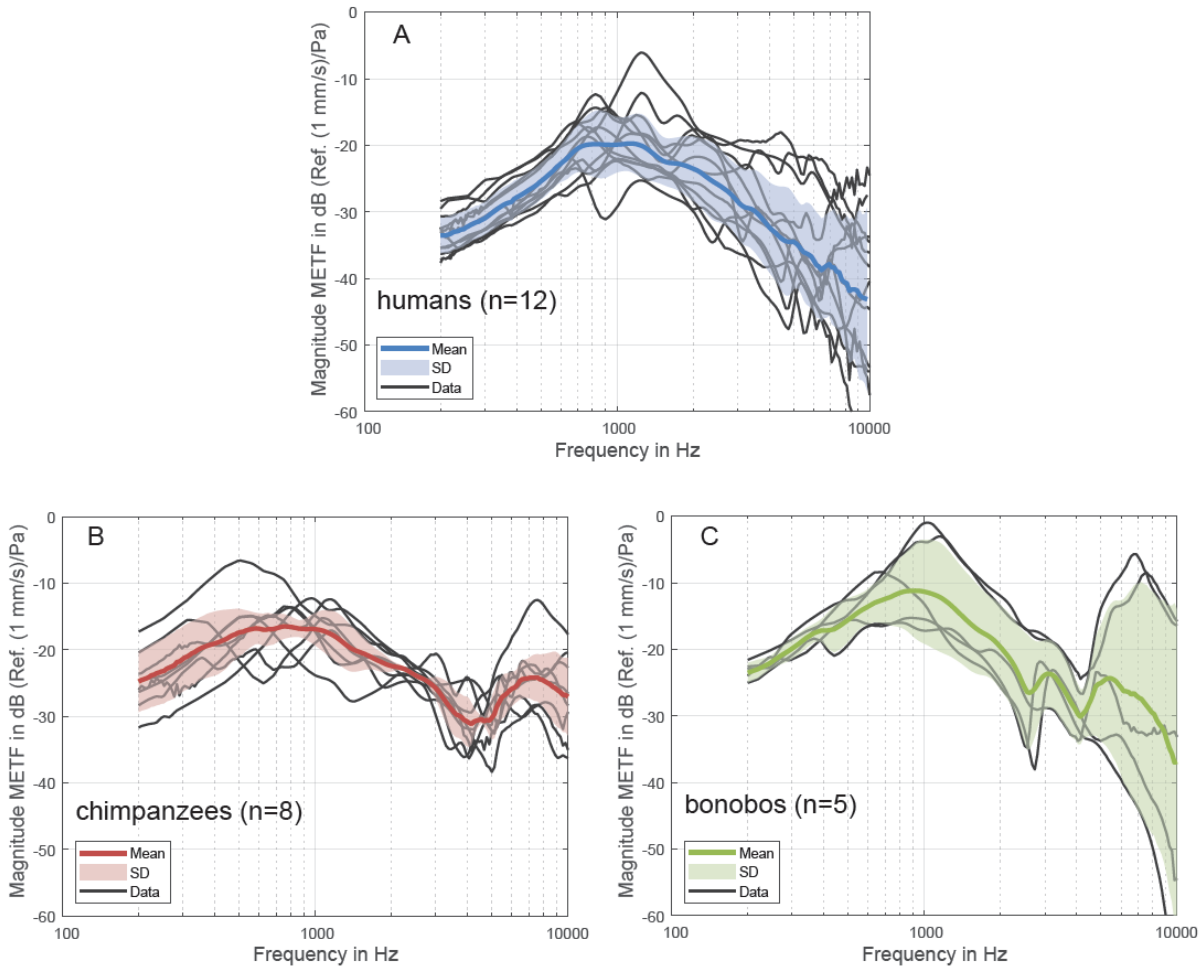

Supplementary Fig. S3. Magnitude of middle ear transfer function of individual ears. (A) *H. sapiens* (blue), (B) *P. troglodytes* (red) (C) *P. paniscus* (green), transparent bar, standard deviation, thick solid lines, mean magnitudes

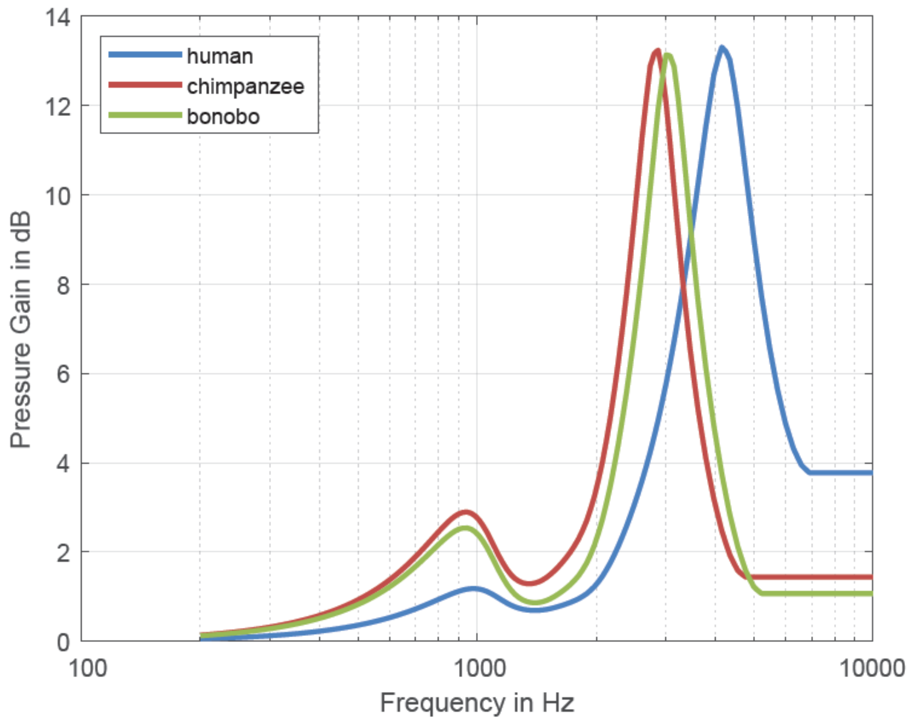

Supplementary Fig. S4. Modelled pressure gain of the external ear canal. *H. sapiens* (blue), *P. troglodytes* (red), *P. paniscus* (green).

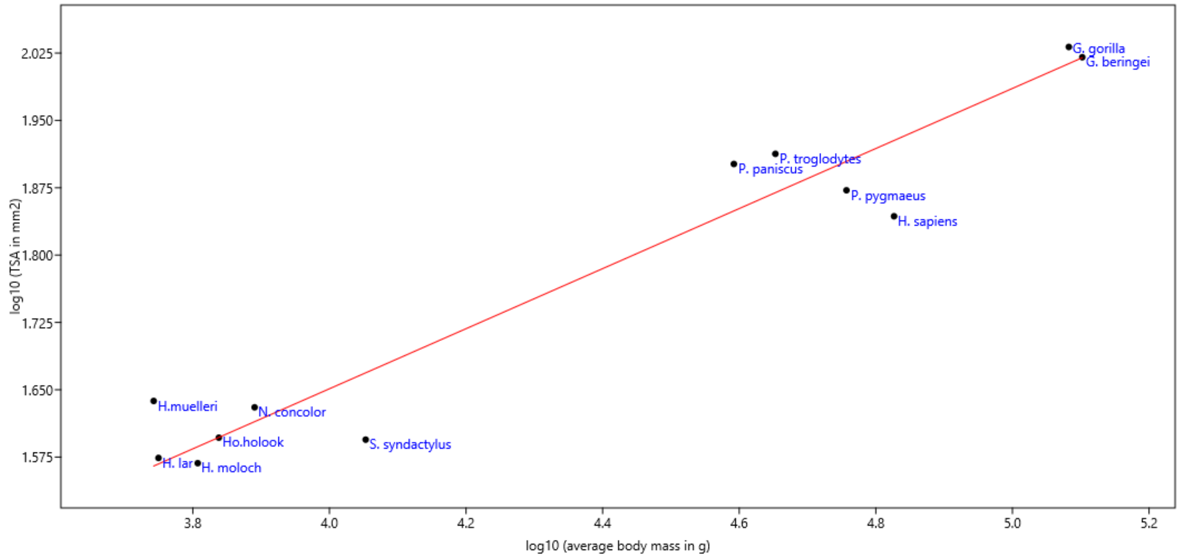

Supplementary Fig. S5. Reduced major axis regression average body mass against the area enclosed by the tympanic sulcus (TSA) of extant hominoid species. Raw data of measurements can be found in the Supplementary Data Table S7; R^2 0.93, p<0.001. (Explanation: to determine phylogenetic polarity of TSA, we measured additional hominoid species and looked at the data relative to body mass. Despite being closest extant relatives, humans and panins differ distinctively in relative tympanic membrane area. Indeed, along with lar gibbons (*Hylobates lar*), chimpanzees and bonobos show the largest relative tympanic membrane areas among all extant hominoids, whereas together with siamangs (*Symphalangus syndactylus*), humans show the smallest. This result points to a strong and opposing selective pressure on tympanic membrane size within the *Pan*/*Homo* clade)

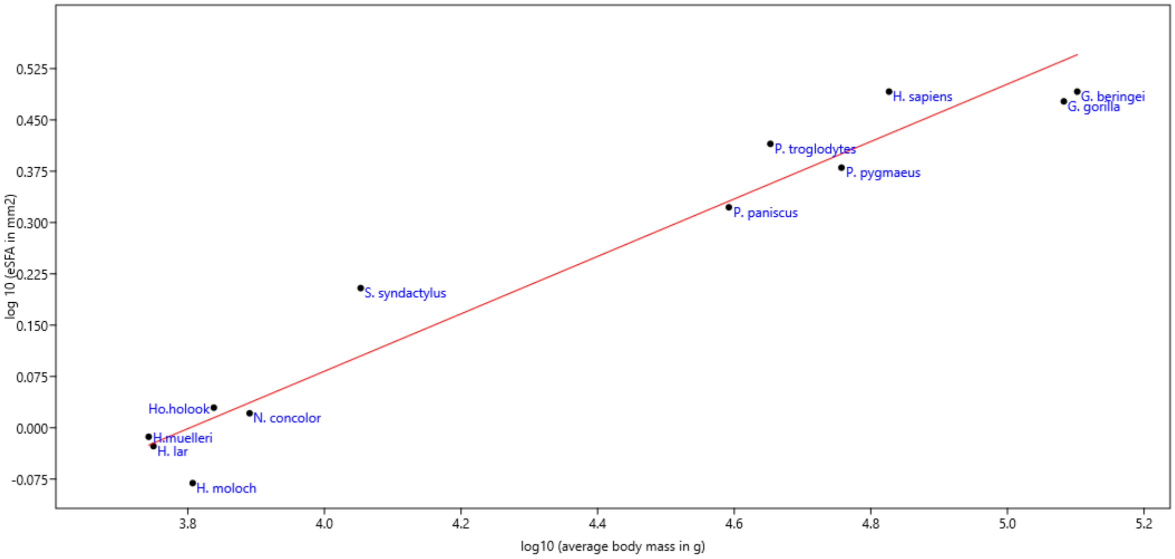

Supplementary Fig. S6 Reduced major axis regression of species' average body mass against the estimated area enclosed by the circumference of the stapes footplate (eSFA) of extant hominoid species. Raw data of measurements can be found in the Supplementary Data Table S7; R2 0.95, p<0.001. Explanation: to determine phylogenetic polarity of eSFA, we measured additional hominoid species and looked at the data relative to body mass. In contrast to TSA, humans share a relatively large stapes footplate area with chimpanzees, but not bonobos, a character state that seems plesiomorphic for the clade.

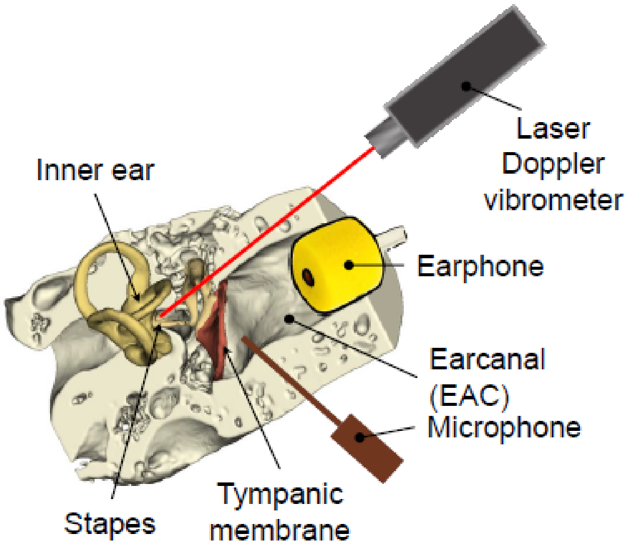

Supplementary Fig. S7. Schematic of the experimental setup for measuring the middle ear transfer function

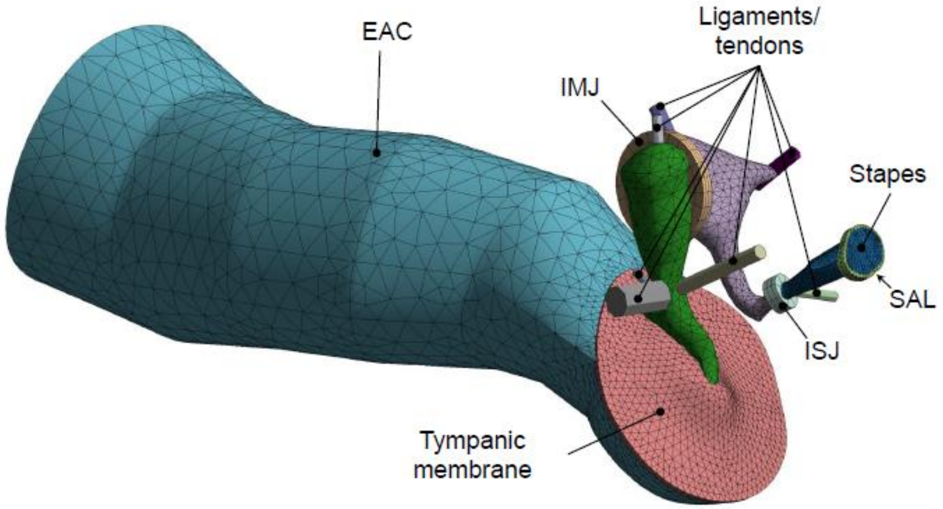

Supplementary Fig. S8. Simulation model for estimating the pressure gain of the external acoustic canal (EEC). IMJ - incudomallear joint, ISJ – incudostapedial joint, SAL – stapededial annular ligament

Supplementary Table S1. Middle ear transfer function (METF).

See attached Dataset file (Excel) Supporting Information Table S1

a.) *H. sapiens*, each measured ear; b.) *H. sapiens*, each individual; c.) *P. troglodytes*, each measured ear; d.) *P. troglodytes* each individual; e.) *P. paniscus*, each ear; f.) *P. paniscus*, each individual; g.) average *Pan* (panins).

Supplementary Table S2. Summary statistics, significance tests and group comparisons of the middle ear transfer function (METF).

See attached Dataset file (Excel) Supporting Information Table S2

a.) statistics of magnitude of first maximum and frequency of first maximum of METF; b.) comparison METF *H. sapiens/P. troglodytes*; c.) comparison METF *H. sapiens/P. paniscus*; d.) comparison METF *P. troglodytes/P. paniscus*; e.) comparison METF *H. sapiens/*panins*.*

Supplementary Table S3. Results of modelled pressure gain of the external acoustic canal (EEC).

See attached Dataset file (Excel) Supporting Information Table S3

Supplementary Table S4. Pressure gain external acoustic canal (EEC).

	**humans**		**chimpanzees**		**bonobos**	
	kHz	dB	kHz	dB	kHz	dB
1. Resonance	4.2	13.3	2.9	13.2	3.0	13.1

Frequency (kHz) and magnitude (dB) of the first external ear canal resonance

Supplementary Table S5. External/middle ear transfer function (EMTF).

See attached Dataset file (Excel) Supporting Information Table S5

a.) *H. sapiens*, all individuals; b.) *P. troglodytes*, all individuals; c.) P. paniscus, all individuals; d.) average panins.

Supplementary Table S6. External/middle ear transfer function (EMTF).

See attached Dataset file (Excel) Supporting Information Table S6

a.) Summary statistics and comparison of key characteristics of the external/middle ear transfer function (EMTF) of humans (Homo), chimpanzees (P. troglodytes), bonobos (P. paniscus) and panins (Pan). Frequency (in kHz), magnitude (in dB) and slopes (dB/octave). Given values are the average and min/max range; b.) & c.) Comparison of significantly different frequency ranges between groups (marked in blue, green corresponds to t-test considered for given frequency) and differences between EMTF key characteristics (first maximum, first minimum, second maximum, second minimum, third maximum and slopes of respective frequency ranges). b.) controlled for the false discovery rate (FDR); c.) without comparison with FDR.

Supplementary Table S7. Summary of morphological parameters of the middle and outer ear.

Taxon	CL (mm)	CMV (mm^3)	TSA (mm^2)	eSFA (mm^2)	Malleus mass (mg)	FL Malleus (mm)	AAF of the malleus (mm^2)	Incus mass (mg)	FL Incus (mm)	AAF of the incus (mm^2)	Stapes mass (mg)	Areas ratio	Lever ratio	ITR	bEECL (mm)	cEECL (mm)	EECL (mm)	EECCS (mm^2)
humans	40.6	65.9	69.7	3.09	22.8	5.52	4.4	26.4	4.60	4.7	2.8	23	1.20	27	16.3	8.1	24.4	72.0
n,	23	1	54	54	25	28	5	26	28	5	22	54	28	27	4	4	4	4
range	36.8-44.5	-	51.6-84.3	2.22-4.21	18.0-29.6	4.96-6.14	4.0-4.8	21.5-31.9	4.27-5.00	4.4-5.1	1.3-3.9	16-31	1.07-1.37	20-43	12.9-18.8	6.5-9.4	19.4-28.2	77.3-93.6
bonobos	39.3	-	79.7	2.08	15.7	6.09	4.5	16.2	3.95	4.5	1.5	38	1.54	60	21.6	10.6	32.2	33.5
n,	6	-	8	8	1	7	3	1	7	3	1	8	7	7	4	4	4	4
range	34.0-42.7	-	71.9-93.4	1.68-2.43	-	5.80-6.40	3.8-5.0	-	3.68-4.12	4.3-4.6	-	34-44	1.48-1.62	52-70	19.2-23.2	9.6-11.6	28.8-34.8	32.4-42.3
chimpanzees	40.8	66.7	81.5	2.62	19.8	6.32	4.6	19.8	3.62	5.0	1.6	31	1.75	53	24.0	12.1	36.1	36.0
n,	8	1	17	17	15	12	5	15	12	5	7	17	12	12	4	4	4	4
range	37.9-42.6	-	68.5-89.3	2.28-2.96	15.0-22.9	5.68-6.85	4.2-5.4	14.2-23.9	3.41-3.79	4.1-6.1	1.3-2.0	24-38	1.62-1.85	42-61	21.8-25.2	10.9-12.6	32.7-37.8	37.0-46.1
gorillas	40.1	61.7	107.6	2.98	20.3	6.36	-	-	4.04	-	-	36	1.57	56	35.1	17.6	52.7	31.0
n,	8	1	10	10	1	8	-	-	8	-	-	10	8	8	11	11	11	4
range	37.5-45.0	-	96.1-133.2	2.43-3.35	-	5.80-6.73	-	-	3.74-4.34	-	-	31-44	1.46-1.70	51-61	27.1-46.3	13.6-23.2	40.7-69.5	27.2-34.1
orangutan	37.9	66.5*	74.5	2.41	17.6	5.71	-	18.3	3.62	-	2.5	31	1.58	49	30.3	15.1	45.4	-
n,	8	1	4	4	7	11	-	7	11	-	2	4	11	4	14	14	14	-
range	32.6-42.1	-	64.4-81.7	2.17-2.63	14.7-19.4	4.95-6.37	-	12.6-25.5	3.10-4.15	-	1.6-3.3	27-38	-	-	21.7-38.0	10.9-19.0	32.6-56.9	-

Values reported correspond to the average and the range observed in studied specimens. CL: Cochlea length, CMV: Volume of the endolymphatic and perilymphatic spaces of the cochlea, TSA: Area enclosed by the tympanic sulcus, eSFA: Estimated stapes footplate area, FL: functional length, AAF: Surface area of the articular facet, ITR: Impedance transformer ratio, bEECL: Bony external ear canal length, cEECL: inferred cartilaginous external ear canal length, EECL: inferred external ear canal length, EECCS: external ear canal cross-section at intermediate position. External ear canal lengths of gorillas and orangutan come from Masalli 1992. * Measured on *Pongo abelii*.

Supplementary Table S8. Summary of specimen IDs, image spatial resolutions of microCT scans and the morphological middle and outer ear parameters of humans, chimpanzees, bonobos and additional hominoid species used in this study.

Species	Specimen	Voxel size (mm)	CL (mm)	CMV (mm^3)	TSA (mm^2)	OWA (mm^2)	eSFA (mm^2)	Malleus mass (mg)	FL Malleus (mm)	AAF of the malleus (mm^2)	Incus mass (mg)	FL Incus (mm)	AAF of the incus (mm^2)	Stapes mass (mg)	Areas ratio	Ossicle lever ratio	ITR	bEECL (mm)	cEECL (mm)	EECL (mm)	EECCS (mm^2)
H. sapiens	Greding-17B	-												3.1							
H. sapiens	Greding-100	0.015<br>0.046			76.51	3.36	3.02		5.30			4.72			25.28	1.12	28.38				
H. sapiens	Greding-101	0.02						24.3			26.4			3.9							
H. sapiens	Greding-113	0.013						21.1			22.6										
H. sapiens	Greding-116	0.015<br>0.046			84.37	2.99	2.69		6.14			4.49			31.35	1.37	42.87				
H. sapiens	Greding-120	0.013<br>0.036			69.36	3.17	2.85	20.8	5.84		23.0	4.56		2.7	24.30	1.28	31.12				
H. sapiens	Greding-121	0.013<br>0.036			70.84	3.53	3.18	22.1	5.99		22.2	4.45		1.3	22.29	1.35	30.01				
H. sapiens	Greding-128	0.015<br>0.04			70.88	2.96	2.66		5.63			4.44			26.63	1.27	33.77				
H. sapiens	Greding-129	0.013<br>0.03			66.22	3.14	2.83	19.4	5.52		25.9	4.64		2.9	23.43	1.19	27.87				
H. sapiens	Greding-131	0.013<br>0.046			76.51	4.11	3.70		5.33			5.00			20.67	1.07	22.03				
H. sapiens	Greding-132	0.013<br>0.04			51.61	3.29	2.96		4.96			4.27			17.42	1.16	20.24				
H. sapiens	Greding-136II	0.013<br>0.046			65.60	3.48	3.13	21.6	5.25		25.4	4.42		2.4	20.97	1.19	24.91				
H. sapiens	Greding-141	0.013<br>0.04			69.02	3.74	3.37	22.6	5.06		28.3	4.56		3.5	20.50	1.11	22.75				
H. sapiens	Greding-144	-												2.4							
H. sapiens	Greding-153	0.013<br>0.04			68.04	3.27	2.94		5.17			4.41			23.10	1.17	27.08				
H. sapiens	Greding-154B	0.02<br>0.04			82.06	3.03	2.73	20.6	5.94	4.0	27.5	4.66	4.6	3.0	30.13	1.27	38.40				
H. sapiens	Greding-162	-						24.6			31.8			3.0							
H. sapiens	Greding-164	-						29.6			29.4										
H. sapiens	Greding-165	-						20.0			24.0										
H. sapiens	Greding-167	0.02<br>0.04			82.20	3.86	3.47	23.3	5.62		27.2	4.48		3.8	23.65	1.25	29.67				
H. sapiens	Greding-180	-						23.6			28.4										
H. sapiens	Greding-184	-						24.1			27.9			3.5							
H. sapiens	Greding-190	-						21.3			25.7										
H. sapiens	Greding-192	-						28.7			31.9			3.1							
H. sapiens	Greding-200	-						27.5			30.0			2.3							

Species	Specimen	Voxel size (mm)	CL (mm)	CMV (mm^3)	TSA (mm^2)	OWA (mm^2)	eSFA (mm^2)	Malleus mass (mg)	FL Malleus (mm)	AAF of the malleus (mm^2)	Incus mass (mg)	FL Incus (mm)	AAF of the incus (mm^2)	Stapes mass (mg)	Areas ratio	Ossicle lever ratio	ITR	bEECL (mm)	cEECL (mm)	EECL (mm)	EECCS (mm^2)
H. sapiens	Greding-203	0.015<br>0.058			74.02	3.96	3.56		5.89			4.35			20.79	1.35	28.15				
H. sapiens	Greding-206	0.015<br>0.046			70.77	3.72	3.35		5.36			4.63			21.16	1.16	24.49				
H. sapiens	Greding-217A	0.013<br>0.035			63.09	3.05	2.75	20.6	5.38	4.1	25.3	4.43	4.4	2.9	22.98	1.21	27.80				
H. sapiens	Greding-22	0.013<br>0.058			76.38	3.38	3.04		5.29	4.6		4.63	4.4		25.13	1.14	28.71				
H. sapiens	Greding-224	0.013			73.17	3.38	3.04		5.48			4.92			24.02	1.11	26.76				
H. sapiens	Greding-225II	0.013<br>0.046			67.77	3.04	2.74	18.0	5.46	4.8	23.6	4.36	5.1	2.6	24.76	1.25	31.00				
H. sapiens	Greding-228	0.013<br>0.058			65.78	2.90	2.61		5.76			4.53			25.23	1.27	32.08				
H. sapiens	Greding-30	0.013<br>0.046			81.16	3.61	3.25		5.79			4.75			24.98	1.22	30.45				
H. sapiens	Greding-38	0.013<br>0.046			67.68	2.46	2.21		5.53			4.83			30.54	1.14	34.97				
H. sapiens	Greding-41A	0.013<br>0.042			70.27	3.09	2.78	21.6	5.39	4.3	21.5	4.49	4.9	2.3	25.24	1.20	30.30				
H. sapiens	Greding-41B	0.013<br>0.058			70.37	3.81	3.43		5.34			4.99			20.51	1.07	21.95				
H. sapiens	Greding-59	-												2.4							
H. sapiens	Greding-66	0.02<br>0.042			81.31	4.01	3.61	23.9	5.64		24.6	4.48		3.9	22.53	1.26	28.37				
H. sapiens	Greding-70	0.013<br>0.058			78.49	3.37	3.03	26.9	5.52		29.7	4.87		3.0	25.89	1.13	29.35				
H. sapiens	Greding-78	0.015<br>0.036			71.24	3.26	2.93		5.33			4.72			24.27	1.13	27.41				
H. sapiens	ULAC-12	0.091	44.2		67.76	3.84	3.46								19.61			16.31	8.16	24.47	71.9
H. sapiens	ULAC-13	0.091	42.6		72.90	3.81	3.43								21.24						
H. sapiens	ULAC-19	0.091	38.5		76.60	3.65	3.29								23.32						
H. sapiens	ULAC-225	0.091	38.8		61.95	2.66	2.39								25.85						
H. sapiens	ULAC-318	0.091	43.8		64.30	3.94	3.55								18.12			12.90	6.5	19.40	70.4
H. sapiens	ULAC-319	0.091	40.6		60.28	2.57	2.31								26.06						
H. sapiens	ULAC-324	0.091	39.1		62.30	3.54	3.19								19.54						
H. sapiens	ULAC-335	0.091	41.2		69.70	2.99	2.69								25.93						
H. sapiens	ULAC-343	0.091	39.1		71.30	3.87	3.48								20.45						
H. sapiens	ULAC-58	0.091	41.2		72.14	3.04	2.74								26.38						
H. sapiens	ULAC-60	0.091	42.6		75.00	3.61	3.25								23.09						
H. sapiens	ULAC-770	0.091	44.5		75.76	3.69	3.32								22.81						

Species	Specimen	Voxel size (mm)	CL (mm)	CMV (mm^3)	TSA (mm^2)	OWA (mm^2)	eSFA (mm^2)	Malleus mass (mg)	FL Malleus (mm)	AAF of the malleus (mm^2)	Incus mass (mg)	FL Incus (mm)	AAF of the incus (mm^2)	Stapes mass (mg)	Areas ratio	Ossicle lever ratio	ITR	bEECL (mm)	cEECL (mm)	EECL (mm)	EECCS (mm^2)
H. sapiens	ULAC-812	0.091	40.7		64.44	3.28	2.95								21.82			18.80	9.4	28.20	78.2
H. sapiens	ULAC-814	0.091	41.3		58.77	3.21	2.89								20.37						
H. sapiens	ULAC-909	0.091	40.5		71.60	3.16	2.84								25.16						
H. sapiens	ULAC-920	0.091	38.2		58.90	3.88	3.49								16.88						
H. sapiens	ULAC-929	0.091			63.30	3.17	2.85								22.21						
H. sapiens	ULAC-955	0.091	36.8		69.02	3.74	3.37								20.49			17.10	8.5	25.6	67.6
H. sapiens	ULAC-959	0.091	39.2		67.30	3.56	3.20								20.98						
H. sapiens	ULAC-961	0.091	37.2		57.10	3.18	2.86								19.95						
H. sapiens	ULAC-978	0.091	39.7		68.03	3.80	3.42								19.88						
H. sapiens	ULAC-985	0.091	39.7		67.20	4.37	3.93								17.11						
H. sapiens	ULAC-996	0.091	41.2		69.22	4.68	4.21								16.44						
H. sapiens	ULAC-1040	0.091	43.0		66.50	3.39	3.05								21.77						
H. sapiens	ULAC G	0.091			61.07	2.69	2.42								25.20						
H. sapiens	UVACinfant13	0.091			70.71	3.71	3.34								21.18						
H. sapiens	UVACinfant23	0.091			74.71	3.36	3.02								24.73						
H. sapiens	HsWOC1	-						21.5			25.3			2.3							
H. sapiens	HsWOC2	-						25.9			28.1										
H. sapiens	HsWOC3	-									25.8			2.1							
H. sapiens	CEB130016	0.0070		65.9																	
P. paniscus	CEB-180009	0.041	37.4		82.6	2.32	2.1		5.8			3.68			39.5	1.58	62				
P. paniscus	CEB-180008	0.041	42.5		80.3	2.02	1.8		6.03			4.02			44.2	1.50	66				
P. paniscus	CEB-180005	0.045			72.6	1.87	1.7	15.7	6.35		16.2	3.91			43.2	1.62	70				
P. paniscus	AMNH-86857	x,v<br>0.058<br>z 0.069	34.0		71.9	2.33	2.1								34.3			19.20	9.6	28.80	32.0
P. paniscus	MAM-3228	0.016	39.4		74.2	2.38	2.1		6.04	5.0		4.02	4.6		34.7	1.50	52				

Species	Specimen	Voxel size (mm)	CL (mm)	CMV (mm^3)	TSA (mm²)	OWA (mm^2)	eSFA (mm²)	Malleus mass (mg)	FL Malleus (mm)	AAF of the malleus (mm²)	Incus mass (mg)	FL Incus (mm)	AAF of the incus (mm²)	Stapes mass (mg)	Areas ratio	Ossicle lever ratio	ITR	bEECL (mm)	cEECL (mm)	EECL (mm)	EECCS (mm²)
P. paniscus	CEB-150021	0.01	40.0		85.9	2.52	2.3		6.14			3.98			37.9	1.54	58				
P. paniscus	CEB-170001	0.011			76.9	2.39	2.1		5.86	4.7		3.97	4.6		35.8	1.48	53				
P. paniscus	CEB-170002	0.015	42.7		93.4	2.70	2.4		6.4	3.8		4.12	4.3		38.4	1.55	60				
P. paniscus	MCZ 38018 M4830-4399	0.0941 08																23.00	11.5	34.50	32.0
P. paniscus	MCZ 38019 M4829-4398	0.0918 94																20.80	10.4	31.20	27.9
P. paniscus	MCZ 38020 M4828-4397	0.0990 81																23.20	11.6	34.80	42.2
P. troglodytes	CEB-130092	0.008			88.7	3.2	2.9		6.56			3.54			31	1.85		57			
P. troglodytes	CEB-130093	0.008			78.2	2.6	2.3		6.81			3.79			33	1.80		59			
P. troglodytes	CEB-140051	0.008			81.5	3.1	2.8		5.68			3.41			29	1.67	49				
P. troglodytes	CEB-180002	-						20.2			19.7										
P. troglodytes	MAM-1306	-						17.8						1.3							
P. troglodytes	MAM-2442	-						21.9			21.5										
P. troglodytes	MAM-2463	0.013 0.092			76.57	2.67	2.40	22.7	6.85	4.9	20.0	3.79	4.6		31.89	1.81	57.72				
P. troglodytes	MAM-2465	-						19.7						1.3							
P. troglodytes	MAM-507	-						15.0			16.9										
P. troglodytes	MAM-512	0.031 0.091			85.98	2.54	2.29	20.3	5.68		21.8	3.5			37.65	1.62	61.10				
P. troglodytes	MAM-7606	0.013								4.2			4.1								
P. troglodytes	MAM-7651	0.013 0.091			78.26	2.66	2.39	19.2	6.06	4.6	18.5	3.46	5.3		32.71	1.75	57.29				
P. troglodytes	MAM-7652	0.013 0.082			83.95	3.10	2.79	17.2			16.5				30.06						
P. troglodytes	TAI-11778	0.069	42.6		87.87	3.11	2.80		6.61			3.65			31.41	1.81	56.88				
P. troglodytes	TAI-11781	0.077	40.1		87.82	3.19	2.87								30.59						
P. troglodytes	TAI-11792*	0.067	40.7		68.47	2.65	2.39		6.25			3.55			28.7	1.76	42.51				
P. troglodytes	TAI-11798	-									20.6										
P. troglodytes	TAI-11800*	0.03 0.069	37.9		75.67	2.62	2.36								32.7						
P. troglodytes	TAI-11903*	0.073	41.7		86.98	3.03	2.73								28.63			24.20	12.1	36.30	39.4

Species	Specimen	Voxel size (mm)	CL (mm)	CMV (mm^3)	TSA (mm^2)	OWA (mm^2)	eSFA (mm^2)	Malleus mass (mg)	FL Malleus (mm)	AAF of the malleus (mm^2)	Incus mass (mg)	FL Incus (mm)	AAF of the incus (mm^2)	Stapes mass (mg)	Areas ratio	Ossicle lever ratio	ITR	bEECL (mm)	cEECL (mm)	EECL (mm)	EECCS (mm^2)
P. troglodytes	TAI-13439	0.015<br>0.069	41.5		72.19	3.29	2.96	22.1	6.41	4.2	20.2	3.69	4.9	2.0	24.35	1.74	42.30				
P. troglodytes	TAI-14991	0.015<br>0.066	39.7		74.75	3.12	2.81	22.9	6.45	4.2	23.6	3.78	4.1		26.58	1.71	45.35				
P. troglodytes	TAI-15011	0.015<br>0.069			89.30	2.83	2.55		6.44			3.75			35.06	1.72	60.21				
P. troglodytes	TAI-15012*	0.079	42.1		82.93	2.78	2.50								33.17			25.22	12.61	37.83	34.4
P. troglodytes	TAI-15020	0.03			86.03	3.02	2.72		6.06			3.57			31.69	1.70	53.80				
P. troglodytes	PtWOC1	-						20.5			18.3			1.5							
P. troglodytes	PtWOCr2	-						15.7			14.2			1.3							
P. troglodytes	PtWOC3	-						21.5			20.4			2.0							
P. troglodytes	PtWOC4	-						20.2			20.9			1.6							
P. troglodytes	MCZ 46416<br>M4815-4384	0.0999<br>18																21.8	10.9	32.7	35.6
P. troglodytes	MCZ 23167<br>M4821-4390	0.1060<br>04																24.9	12.5	37.4	34.4
P. troglodytes	CEB-140051	0.0079		67.3																	
G. gorilla	MAM-4531	0.013<br>0.086			118.10	3.26	2.93		6.35			4.17			40.29	1.52	61.35				
G. gorilla	MAM-469	0.013			96.63	3.27	2.94								32.86						
G. gorilla	MAM-493	0.082			107.44	2.70	2.43								44.21						
G. gorilla	MAM-7644	0.037<br>0.091			109.91	3.48	3.13		5.8			3.97			35.07	1.46	51.24				
G. gorilla	ZMB-10493	0.027			101.20	3.50	3.15		6.36			3.74			32.15	1.70	54.66				
G. gorilla	ZMB-11642	0.2129,<br>0.2129,<br>0.335	38.1																		
G. gorilla	ZMB-11683	0.027			111.52	3.31	2.98		6.67			4.07			37.42	1.64	61.33				
G. gorilla	ZMB-12206	0.208<br>0.208,<br>0.335	39.4																		
G. gorilla	ZMB-14644	0.2129,<br>0.2129,<br>0.335	37.9																		
G. gorilla	ZMB-14645	0.224<br>0.224,<br>0.335	40.8																		
G. gorilla	ZMB-14647	0.195<br>0.195,<br>0.335	41.2																		
G. gorilla	ZMB-17802	0.064	45.0																		

G. gorilla	ZMB-20305	0.03			133.24	3.72	3.35		6.34			4.11			39.83	1.54	61.44				
G. gorilla	ZMB-31229	0.027	40.5		103.18	3.24	2.92		6.32			4.06			35.42	1.56	55.13				
Species	Specimen	Voxel size (mm)	CL (mm)	CMV (mm^3)	TSA (mm^2)	OWA (mm^2)	eSFA (mm^2)	Malleus mass (mg)	FL Malleus (mm)	AAF of the malleus (mm^2)	Incus mass (mg)	FL Incus (mm)	AAF of the incus (mm^2)	Stapes mass (mg)	Areas ratio	Ossicle lever ratio	ITR	bEECL (mm)	cEECL (mm)	EECL (mm)	EECCS (mm^2)
G. gorilla	ZMB-31435	0.027	37.5		98.47	3.27	2.94		6.73			4.34			33.42	1.55	51.82				
G. gorilla	ZMB-6980	0.029			96.05	3.40	3.06		6.3			3.87			31.40	1.63	51.11				
G. gorilla	CEB-140059	0.0079		61.7																	
G. beringei	ZMB-31617	0.021			130.9	3.74	3.37								38.88						
G. beringei	ZMB-31622	0.028			99.3	3.38	3.04								32.64						
G. beringei	ZMB-37523	0.064			84.1	3.13	2.82								29.84						
P.pygmaeus	ZMB-6954	0.149	37.0																		
P.pygmaeus	ZMB-6957	0.149	38.8																		
P.pygmaeus	ZMB-6973	0.026	32.6		81.7	2.41	2.17								37.66						
P.pygmaeus	ZMB-6978	0.026			64.4	2.55	2.29								28.09						
P.pygmaeus	ZMB-7875	0.091	37.9																		
P.pygmaeus	ZMB-8007	0.175 0.175, 0.335	37.7																		
P.pygmaeus	ZMB-13255	0.152 0.152, 0.335	36.3																		
P.pygmaeus	ZMB-30945	0.182 0.182, 0.335	41.0																		
P.pygmaeus	ZMB-33670	0.195 0.195, 0.335	42.1																		
P.pygmaeus	ZMB-83501	0.026			70.1	2.93	2.63								26.61						
P.pygmaeus	ZMB-87086	0.026			81.6	2.85	2.56								31.88						
Pongo sp.	A89,04							19.1			22.9										
Pongo sp.	7876							17.9			18.3										
Pongo sp.	A208,12							19.0			25.5			1.6							
Pongo sp.	11257							15.8			12.6			3.3							
Pongo sp.	no id							17.5			18.7										

Pongo sp.	6947							19.4			17.5										
Pongo sp.	166							14.7			12.8										
Species	Specimen	Voxel size (mm)	CL (mm)	CMV (mm^3)	TSA (mm^2)	OWA (mm^2)	eSFA (mm^2)	Malleus mass (mg)	FL Malleus (mm)	AAF of the malleus (mm^2)	Incus mass (mg)	FL Incus (mm)	AAF of the incus (mm^2)	Stapes mass (mg)	Areas ratio	Ossicle lever ratio	ITR	bEECL (mm)	cEECL (mm)	EECL (mm)	EECCS (mm^2)
P. abelii	CEB-140065	0.0079		66.5																	
S. syndactylus	ZMB-22607	0.017			42.2	1.63	1.47								28.66						
S. syndactylus	ZMB-7850	0.017			35.1	1.86	1.67								20.95						
S. syndactylus	ZMB-38582	0.017			40.6	1.77	1.59								25.47						
N. concolor	ZMB-7803	0.064			42.7	1.17	1.05								40.58						
H. hoolock	ZMB-7837	0.016			39.5	1.19	1.07								37.06						
H.lar	ZMB-42605	0.036			37.5	1.05	0.94								39.83						
H. moloch	ZMB-67172	0.063			37.0	0.92	0.83								44.48						
H.muelleri	ZMB-85392	0.063			43.4	1.08	0.97								44.53						

Abbreviations: CL: Cochlea length, CMV: Volume of the endolymphatic and perilymphatic spaces of the cochlea, TSA: Area enclosed by the tympanic sulcus, eSFA: Estimated stapes footplate area, FL: functional length, AAF: Surface area of the articular facet, ITR: Impedance transformer ratio, bEECL: Bony external ear canal length, cEECL: Inferred cartilaginous external ear canal length, EECL: inferred external ear canal length, EECCS: average external ear canal cross-section. AMNH, American Museum of Natural History; CEB, Comparative Ear Bank collection housed at Max Planck Institute for Evolutionary Anthropology, Leipzig; Greding, collection of medieval graves from Greding, Germany housed at University Hildesheim; MAM, Mammal Collection Phyletisches Museum, Friedrich-Schiller-Universität Jena;; CEB, MPI EVA, Max Planck Institute for Evolutionary Anthropology; TAI, Taï chimpanzee collection housed at Max Planck Institute for Evolutionary Anthropology; ULAC, University of Leipzig Anatomy collection; UVAC, University of Vienna anatomical collection; WOC, Werner Ossicle Collection; WZS, Wilhelma Zoological Garden Stuttgart; ZMB, Zoological collection of the Museum für Naturkunde Berlin; *value has changed from previously published. If two values are present in cells giving voxel sizes, then ossicles and temporal bone/skull were scanned separately: first value resolution, ossicles; second value resolution, the temporal/skull.

References:

Masali, M., Tarli, S. B., & Maffei, M. (1992). Auditory ossicles and the evolution of the primate ear: a biomechanical approach. Language origin: A multidisciplinary approach, 67-86.

Table S9 Species, method for obtaining data and original source of primate audiograms used in this study

Taxon	Type	Reference
Aotus trivirgatus	Speakers	Beecher 1974a
Callithrix jacchus	Speakers	Seiden 1958, Osmanski & Wang 2011
Carlito syrichta	ABR	Ramsier et al. 2012b
Cercopithecus neglectus	Headphones	Owren et al. 1988
Chlorocebus aethiops	Headphones	Owren et al. 1988
Daubentonia madagascariensis	ABR	Ramsier et al. 2012a
Eulemur coronatus	ABR	Ramsier et al. 2012a
Eulemur fulvus	ABR	Ramsier et al. 2012a
Eulemur mongoz	ABR	Ramsier et al. 2012a
Eulemur rubriventer	ABR	Ramsier et al. 2012a
Galago senegalensis	Speakers	Heffner et al. 1969
Homo sapiens	Speakers	ISO 226 2003
Lemur catta	Speakers	Gillette et al. 1973
Lemur catta	ABR	Ramsier et al. 2012a
Macaca fascicularis	Speakers	Fujita & Elliott 1965
Macaca fascicularis	Headphones	Stebbins et al. 1966
Macaca fuscata	Speakers	Jackson et al. 1999
Macaca fuscata	Headphones	Owren et al. 1988
Macaca mulatta	Speakers	Pfingst et al. 1978, Lonsbury-Martin & Martin 1981, Coleman 2009
Macaca nemestrina	Headphones	Coleman 2009
Microcebus murinus	ABR	Schopf et al. 2014
Nycticebus coucang	Speakers	Heffner & Masterton 1970
Nycticebus coucang	ABR	Ramsier et al. 2012a
Nycticebus pygmaeus	ABR	Ramsier et al. 2012a
Pan troglodytes	Headphones	Kojima 1990, Elder 1934
Papio cynocephalus	Speakers	Hienz et al. 1982
Perodicticus potto	Speakers	Heffner & Masterton 1970
Phaner furcifer	Speakers	Niaussat & Molin 1978
Propithecus coquereli	ABR	Ramsier et al. 2012a
Saimiri sciureus	Speakers	Beecher 1974b
Sapajus apella	ABR	Ramsier et al. 2017
Varecia rubra	ABR	Ramsier et al. 2012a

References:

Beecher, M.D. (1974a) Hearing in the owl monkey (aotus trivirgatus). Journal of Comparative and Physiological Psychology, 86, 898-901.

Beecher, M.D. (1974b) Pure tone thresholds of the squirrel monkey (Saimir sciureus). Journal of the Acoustical Society of America, 55, 196-198.

Coleman, M.N. (2009) What Do Primates Hear? A Meta-analysis of All Known Nonhuman Primate Behavioral Audiograms. International Journal of Primatology, 30, 55-91.
Fujita, S. & Elliott, D. (1965). Thresholds of audition in three species of monkey. The Journal of the Acoustical Society of America, 37, 139–144.
Gillette, R.G., Brown, R., Herman, P., Vernon, S. & Vernon, J. (1973) The auditory sensitivity of the lemur. American Journal of Physical Anthropology, 38, 365-370.
Heffner, H., & Masterton, B. (1970) Hearing in primitive primates: Slow loris (Nycticebus coucang) and potto (perodicticus potto). Journal of Comparative and Physiological Psychology, 71, 175-182.
Heffner, H.E., Ravizza, R.J. & Masterton, B.(1969) Hearing in primitive mammals, IV: Bushbaby. Journal of Auditory Research, 9, 19-23.
Hienz, R.D., Turkkan, J.S. & Harris, A.H. (1982) Pure tone thresholds in the yellow baboon (Papio cynocephalus). Hearing Research, 8, 71-75.
ISO 226 (2003). Acoustics – normal equal loudness level contours.
Jackson, L.S., Heffner, R.S. & Heffner, H.E. (1999) Free-field audiogram of the Japanese macaque (Macaca fuscata). Journal of the Acoustical Society of America, 106, 3017-3023.
Kojima, S. (1990) Comparison of Auditory Functions in the Chimpanzee and Human. Folia Primatologica, 55, 62-72.
Lonsbury-Martin, B. & Martin, G. (1981) Effects of moderately intense sound on auditory sensitivity in rhesus monkeys: Behavioral and neural observations. Journal of Neurophysiology, 46, 563-586.
Niaussat, M-.M., & Molin, D. (1978). Hearing and vocalization in a Malagasy lemur: Phaner furcifer. In Recent advances in primatology, Vol. 1: Behavior (ed Chivers, D. J.), pp. 821–825. London: Academic Press.
Osmanski, M.S. & Wang, X. (2011) Measurement of absolute auditory thresholds in the common marmoset (Callithrix jacchus). Hearing Research, 277, 127-133.
Owren, M.J., Hopp, S.L., Sinnott, J.M. & Petersen, M.R. (1988) Absolute auditory thresholds in three old world monkey species (Cercopithecus aethiops, C. neglectus, Macaca fuscata) and humans (Homo sapiens). Journal of Comparative Psychology, 102, 99-107.
Pfingst, B.E., Laycock, J., Flammino, F., Lonsbury-Martin, B. & Martin, G. (1978) Pure tone thresholds for the rhesus monkey. Hearing Research, 1, 43-47.
Ramsier, M.A., Cunningham, A.J., Finneran, J.J. & Dominy, N.J. (2012a) Social drive and the evolution of primate hearing. Philosophical Transactions of the Royal Society B, 367, 1860-1868.
Ramsier, M.A., Cunningham, A.J., Moritz, G.L., Finneran, J.J., Williams, C.V., Ong, P.S., Gursky-Doyen, S.L. & Dominy, N.J. (2012b) Primate communication in the pure ultrasound. Biology Letters, 8 (4). doi:10.1098/rsbl.2011.1149
Schopf, C., Zimmermann, E., Tunsmeyer, J., Kastner, S.B.R., Hubka, P. & Kral, A. (2014) Hearing and Age-Related Changes in the Gray Mouse Lemur. Journal of the Association for Research in Otolaryngology, 15, 993-1005.
Seiden, H.R. (1958) Auditory acuity of the marmoset monkey (Hapale jacchus). Unpublished Doctoral Dissertation, Princeton University.
Stebbins, W.C., Green, S. & Miller, F.L. (1966) Auditory sensitivity of the monkey. Science, 153, 1646-1647.

Table S10. Model parameters for FEA analysis pressure gain of the external ear canal (EEC).

See attached Dataset file (Excel) Supporting Information Table S9
